# Microbiota and lipid mediators: from molecular crosstalk to therapeutic opportunities

**DOI:** 10.3389/fphar.2026.1836931

**Published:** 2026-07-16

**Authors:** Büşra Başar Gökcen, Büşra Atabilen Pınar, Burcu Deniz Güneş, Merve Esra Çıtar Dazıroğlu, Şehriban Duyar Özer, Aziz Alper Biten, Ester Pagano, Maria Francesca Nanì, Barbara Romano, Duygu Ağagündüz, Raffaele Capasso

**Affiliations:** 1 Department of Nutrition and Dietetics, Fethiye Faculty of Health Sciences, Muğla Sıtkı Koçman University, Muğla, Türkiye; 2 Department of Nutrition and Dietetics, Faculty of Health Sciences, Karamanoğlu Mehmetbey University, Karaman, Türkiye; 3 Department of Nutrition and Dietetics, Faculty of Health Sciences, Aydın Adnan Menderes University, Aydın, Türkiye; 4 Department of Nutrition and Dietetics, Faculty of Health Sciences, Gazi University, Ankara, Türkiye; 5 Department of Nutrition and Dietetics, Faculty of Health Sciences, Hitit University, Çorum, Türkiye; 6 European Union and Foreign Affairs, Republic of Turkey Ministry of Health, Ankara, Türkiye; 7 Department of Pharmacy, University of Naples Federico II, Naples, Italy; 8 Department of Agricultural Sciences, University of Naples Federico II, Naples, Italy

**Keywords:** bile acids, gut microbiota, lipid mediators, lipid metabolism, metabolic diseases, short-chain fatty acids

## Abstract

The gut microbiota constitutes a metabolically active ecosystem that dynamically interacts with the host lipid metabolism and cellular signaling networks. While dietary lipids shape the microbial composition and metabolite production, the microbiota reciprocally biotransform fatty acids, thereby regulating lipid mediator bioavailability and host cellular responses. This bidirectional molecular crosstalk plays a pivotal role in maintaining metabolic homeostasis, epithelial barrier integrity, and immune regulation. Microbiota-derived lipid signals, including short-chain fatty acids, bile acids, polyunsaturated fatty acid-derived intermediates, sphingolipids, and N-acyl amides, modulate the balance between pro-inflammatory eicosanoids and specialized-pro resolving mediators. Through the engagement of G protein-coupled receptors, nuclear receptors such as Farnesoid X receptor and peroxisome proliferator activated receptors, Takeda G protein-coupled receptor 5, and the endocannabinoid system, these molecules influence gene expression, cytokine production, and cellular proliferation, thereby shaping the host physiology. Microbial enzymatic transformations, particularly bile acid biotransformation and fatty acid epoxide metabolism, further remodel the lipid mediator network and its biological effects. Disruption of this regulatory axis is associated with increased intestinal permeability, chronic low-grade inflammation, and metabolic dysfunction, contributing to the pathogenesis of metabolic syndrome, non-alcoholic fatty liver disease, inflammatory bowel diseases, and cardiometabolic disorders. From a therapeutic perspective, multicomponent interventions integrating probiotics, prebiotics, post-biotics, dietary modulation, and physical activity may rebalance the lipid mediator profiles and attenuate inflammatory burden. In this context, the microbiota–lipid mediator interface emerges as a translationally relevant biological target for the prevention and management of chronic diseases.

## 1 Introduction and conceptual framework

### 1.1 Gut microbiota: structural organization, functional roles, and systemic impact

The human gastrointestinal tract harbors a complex gut microbiota, with the colon containing the most dense and metabolically active microbial ecosystem (Ma and Lee, 2025). The human colon contains approximately 10 times more microbial cells than the rest of the body. This corresponds to nearly 100 trillion microorganisms and approximately 500–1,500 microbial species per individual, forming a total biomass of approximately 2 kg. Recent metagenomic and culture-based studies have demonstrated that the human gut microbiota harbors substantial taxonomic and genetic diversity, although inter-individual microbial composition varies considerably across populations and geographic regions (Feng et al., 2022; Beam et al., 2021; Ma et al., 2026; Almeida et al., 2021; Andreu-Sánchez et al., 2025).

Despite the taxonomic diversity of the gut microbiota, encompassing bacteria, fungi, viruses, archaea, and microeukaryotes, the current literature remains largely centered on bacterial communities (Fang et al., 2024; Perler et al., 2023). *Bacteroidetes*, *Firmicutes*, *Actinobacteria*, *Proteobacteria*, and *Verrucomicrobia* are the five primary phyla of bacteria found in the human gut. *Bacteroidetes* and *Firmicutes* are the two most important phyla, accounting for more than 90% of microbial abundance in a healthy gut ecosystem (Zhang, 2022; Chandrasekaran et al., 2024). This community’s metabolic activity is vital in maintaining and regulating the host’s physiological homeostasis (Pires et al., 2024). By producing short-chain fatty acids (SCFAs) and attenuating the inflammatory responses, beneficial bacterial genera such as *Lactobacillus* and *Bifidobacterium*, which belong to the *Firmicutes* and *Actinobacteria* phyla, respectively, contribute to the maintenance of gut health. On the other hand, increased concentrations of some *Proteobacteria* species, such as *Desulfovibrio* and *Escherichia coli*, have been linked to metabolic problems and inflammatory processes (Mohammadi and Rudkowska, 2025).

The gut microbiota possesses the capacity to carry out various metabolic processes that cannot be performed independently by the host but are essential for maintaining physiological homeostasis (Van Hul et al., 2024). In this context, it serves as a key determinant of local metabolic functions, including nutrient digestion and absorption, energy extraction from dietary components, elimination of metabolic end-products, and the regulation of intestinal permeability, motility, and hormone secretion (Olofsson and Bäckhed, 2022). In addition to its metabolic functions, the gut microbiota serves as a source of diverse biomolecules with immunomodulatory, cytotoxic, antioxidant, and antibacterial properties (Wadan et al., 2025). Owing to these multifaceted activities, the gut microbiota is increasingly conceptualized as a “living organ,” characterized by its integration into a dynamic inter-organ communication network operating through neural, endocrine, humoral, immune, and metabolic signaling pathways. This perspective echoes Hippocrates’ well-known statement from 400 BC that “death sits in the intestines” (Afzaal et al., 2022).

### 1.2 Lipid mediators: classification, biosynthesis, and receptor systems

Lipids have historically been linked to two main biological roles: (1) acting as sources of metabolic energy and (2) acting as crucial structural elements that maintain the integrity of cellular membranes. Beyond their structural contribution to cellular membranes, lipids are now recognized as bioactive molecules with essential roles in signal transduction and cellular regulation. This early conceptual shift laid the foundation for the extensive study of a wide range of fatty acid-derived bioactive lipid mediators as regulatory signaling molecules in physiological and pathophysiological processes (Dyall et al., 2022).

The release of polyunsaturated fatty acids (PUFAs) from various lipid fractions initiates the synthesis of lipid mediators, which are potent bioactive signaling molecules with multifaceted roles in modulating and regulating immune and inflammatory responses (Werz, 2023). These lipid mediators constitute a prominent category of bioactive metabolites within the organism (Nishi et al., 2021). Based on their structures and functions, lipid mediators are classified into three principal groups, namely, eicosanoids, lysophospholipids, and omega-3 PUFA derivatives (Lubrano et al., 2023). Phospholipase A_2_ (PLA_2_) releases arachidonic acid (AA), eicosapentaenoic acid (EPA), and docosahexaenoic acid (DHA) from membrane phospholipids during cellular stimulation, which is the main source of these chemicals (Zhang K. et al., 2021). Lysophospholipids and free fatty acids are produced when PLA_2_ hydrolyzes phospholipids at the sn-2 location. Through the activities of cyclooxygenases (COX), lipoxygenases (LOX), and cytochrome P450 (CYP) enzymes, along with through interactions with certain receptors, such as G protein-coupled receptors (GPCRs), the released fatty acids are subsequently transformed into a wide range of lipid mediators (Nishi et al., 2021; Zhang K. et al., 2021; Torres and Cyster, 2023).

The effects of these lipid mediators vary depending on the type of PUFAs from which they are derived, along with the enzymes and receptors involved in their biosynthesis and signaling (Lubrano et al., 2023). These mediators play key roles in initiating acute inflammatory responses or promoting their resolution (Kytikova et al., 2019). AA acts as a key substrate for the biosynthesis of inflammatory lipid mediators, including prostaglandins (PGs), leukotrienes (LTs), and hydroxyeicosatetraenoic acids (HETEs), which together constitute the eicosanoid family. In contrast, omega-3 fatty acids such as EPA and DHA serve as precursors for specialized pro-resolving mediators (SPMs), including resolvins (Rvs), protectins, and maresins, which have significant anti-inflammatory and inflammation-resolving activities (Murakami et al., 2022). These pro-resolving lipid mediators actively promote the resolution phase of inflammation through several mechanisms: limiting neutrophil infiltration into tissues, enhancing efferocytosis (phagocytic clearance of apoptotic cells), supporting pathogen elimination, and simultaneously decreasing the synthesis and secretion of pro-inflammatory mediators (Derada Troletti et al., 2021).

### 1.3 Interconnectivity between diet, gut microbiota, and lipid metabolism

Microbial metabolism of carbohydrates and proteins has been extensively studied; however, earlier studies predominantly focused on water-soluble polar metabolites, leading to a relative under-exploration of the microbiota–host–lipid axis (Huyan et al., 2022; Lamichhane et al., 2021). However, the potential systemic effects of lipids produced by the gut microbiota, particularly their ability to cross the epithelial barrier and enter circulation due to their lipophilic nature, have increasingly gained attention as an emerging area of interest (Trošt et al., 2020).

The gut microbiota synthesizes a vast array of enzymes with versatile capabilities to ferment compounds that escape digestion by human enzymes (Jyoti and Dey, 2025; Liu J. et al., 2022). With this extensive enzymatic repertoire, the microbiota is also capable of degrading, remodeling, and detoxifying dietary lipids, which are functions that collectively allow it to act as a ‘secondary liver’ (Brown et al., 2023). Beyond lipid substrates, the gut microbiota can generate lipid-related metabolites from non-lipid dietary inputs through microbial fermentation processes. Microbial fermentation of carbohydrates and proteins produces SCFAs and branched-chain fatty acids (BCFAs), among which acetate, propionate, and butyrate are the most abundant in the intestinal environment (Bourdeau-Julien et al., 2023). The relative abundance of these metabolites varies across the bacterial taxa, with acetate and propionate primarily associated with *Bacteroides* species, butyrate production linked to *Faecalibacterium prausnitzii*, and acetate generation commonly observed among *Lactobacillus* species (Mohammadi and Rudkowska, 2025). Among SCFAs, butyrate is largely retained within the colonic lumen, where it serves as a principal energy substrate for intestinal epithelial cells and contributes to the maintenance of epithelial barrier integrity. Butyrate also regulates gene expression related to cell proliferation, differentiation, and inflammatory responses through the inhibition of histone deacetylases (HDACs). In addition, SCFAs function as signaling molecules *via* the activation of GPCRs expressed on intestinal epithelial and immune cells (Olofsson and Bäckhed, 2022; Wang L.-Y. et al., 2024).

BCFAs are formed in the gut *via* microbial processing of BCFAs, particularly valine, leucine, and isoleucine, and they include isobutyric, isocaproic, and isovaleric acids. This fermentation pathway is carried out primarily by bacterial genera such as *Bacteroides*, *Propionibacterium*, and *Clostridium* (Mohammadi and Rudkowska, 2025; Rios-Covian et al., 2020). Although BCFAs incorporated into microbial membrane lipids have been shown to influence immune homeostasis (Lu H. et al., 2024), the physiological implications of these fatty acids for the overall host health remain largely unclear (Li et al., 2025).

In contrast to lipids synthesized by the host, bacterial lipids frequently display distinctive features, including odd-numbered fatty acid chains, expanded variability in saturation and desaturation patterns, and a broad diversity of polar head group structures (Van Hul et al., 2024). From a quantitative standpoint, lipids constitute a substantial fraction of the gut microbiome’s overall metabolic output, underscoring their central role in microbial physiology and host–microbe interactions (Nguyen et al., 2025). Bacterial lipids play a critical role in fundamental biological processes such as maintaining the structural integrity of the cell membrane, sustaining energy production *via* the electron transport chain, functional assembling membrane proteins, and protecting the cell against environmental stressors. Each bacterial species has a unique lipid profile reflecting its genetically determined biosynthetic pathways and adaptation to its ecological niche (Brown et al., 2023).

The diversity of lipids produced by bacteria is remarkably extensive; however, much of this diversity within the gut microbiota remains unresolved. A substantial proportion of the lipids detected in fecal samples through metabolomic analyses are still unidentified, and their biological functions remain unknown. Consequently, recent research has increasingly focused on elucidating these previously uncharacterized lipids within the bacterial lipidome (Brown et al., 2023). The compositional profile of the human fecal lipidome was first characterized by Gregory and colleagues, who identified more than 500 intact lipid species corresponding to six of the eight major categories defined by the LIPID MAPS classification. These included:

1. Glycerophospholipids (phosphocholine, phosphoethanolamine, phosphoserine, phosphoinositol, phosphoglycerol, and phosphatidic acid),
2. Fatty acids,
3. Sphingolipids (sphingomyelins, ceramides, and gangliosides),
4. Glycerolipids (diacylglycerols and triacylglycerols),
5. Sterol lipids (bile acids and derivatives and cholesterol esters), and
6. Prenol lipids (coenzyme Q) (Gregory et al., 2013).

The production of specific lipid classes by distinct bacterial phyla highlights the ‘lipid mediator’ dimension of microbiota–host interactions. Many of these lipid classes exhibit phylum- or taxon-specific distribution patterns. For example, members of the phylum *Bacteroidetes*, which account for approximately 30%–40% of the gut microbiota and include genera such as *Bacteroides*, *Prevotella*, and *Porphyromonas*, are capable of synthesizing unique sphingolipids that modulate host lipid metabolism. Notably, the gut microbiota possesses serine palmitoyl transferase, the enzyme responsible for the first step of *de novo* sphingolipid synthesis (Johnson et al., 2020; Lee et al., 2021; Heaver et al., 2018). Similarly, saccharolipids, which consist of acylated lipid moieties linked to sugar head groups of varying sizes, most notably lipopolysaccharides (LPS) and lipooligosaccharides, are produced by a wide range of Gram-negative bacteria (Kim et al., 2024; Zhang et al., 2024). Moreover, a subset of bacterial species within the gut microbiome has been shown to synthesize plasmalogens, a class of glycerophospholipids characterized by a vinyl ether bond instead of an ester bond. Plasmalogens are thought to play roles in protecting cells from oxidative stress and in both the initiation and resolution of chronic inflammation (Brown et al., 2023). In addition, microorganisms contribute to the host lipid homeostasis by participating in hepatic lipid metabolic pathways, including the elongation and desaturation of PUFAs (Kumar et al., 2025). Furthermore, species of *Lactobacillus* and *Bifidobacterium* are known to assimilate cholesterol and, in some cases, reduce its intestinal absorption, thereby modulating the host cholesterol levels (Nguyen et al., 2025).

These findings demonstrate that the gut microbiota contributes to dietary lipid processing while simultaneously functioning as a biosynthetic system that generates structurally distinct lipid molecules. Compared with host-derived lipids, microbial lipids differ in acyl-chain characteristics, stereochemistry, and polar head group composition, enabling them to modulate the host physiology through interactions with diverse receptor systems. Subtle structural variations in these lipids can markedly influence receptor signaling, underscoring the importance of characterizing microbial lipid structures to understand their role in host metabolic and immune homeostasis (Morozumi et al., 2022). Therefore, fully characterizing microbial lipid diversity and defining its impact on host biology remain critical requirements for advancing research on the microbiota–lipid axis.

A summary of microbiota-derived lipids, including their structures, microbiome sources, receptors, and biological effects, is presented in Table 1.

**TABLE 1:** Classification of microbiota-associated lipids and lipid mediators according to their structural features, microbial sources, receptor targets, and biological functions.

Category	Lipid class/Subtype	Microbial source/Association	Main receptor/Pathway	Major biological effects
Dietary lipids	n-3 LC PUFAs	Diet–microbiota interaction; modulation of *Firmicutes/Bacteroidetes ratio*, *Lactobacillus*, *Bifidobacterium*, Enterobacteriaceae*, Akkermansia,* and *Faecalibacterium*	NF-κB, NLRP3 inflammasome, MAPK (ERK/JNK), PPARα/γ, GPR120, GPR40, and TGF-β signaling pathway	Modulation of gut bacterial composition, anti-inflammatory effects, improved intestinal barrier integrity, and reduced endotoxemia and intestinal inflammation
	n-6 LC PUFAs			Pro-inflammatory effects, increased intestinal permeability, and altered gut microbiota composition
Microbiota-derived lipid metabolites	Short-chain fatty acids	*Lactobacillus*, *Bifidobacterium*, *Prevotella,* and *Bacteroides*	FFAR2 (GPR43), FFAR3 (GPR41), and GPR109A (HCAR2) signaling pathway	Anti-inflammatory activity, anti-oxidant activity, maintenance of intestinal barrier integrity, and regulation of metabolic and inflammatory responses
	Secondary bile acids	*Bacteroides*, *Clostridium*, *Lactobacillus*, and *Bifidobacterium*	TGR5, FXR, VDR, PXR, and CAR signaling pathway	Regulation of glucose and lipid metabolism, immune modulation, and maintenance of intestinal homeostasis
	PUFA-derived intermediate metabolites	*Lactobacillus plantarum, Butyrivibrio fibrisolvens, Lactococcus lactis,* and *Bifidobacterium*	Ras/Raf/MAPK (MEK)/ERK signaling pathway	Anti-inflammatory effects, suppression of pathogen colonization, and modulation of host metabolism and immune responses
Structural microbial lipids	Membrane lipids (cholesterol, sphingolipids, gangliosides, *etc.*)	*Bacteroides*, *Porphyromonas*, *Prevotella*, *Flectobacillus*, and *Chlorobium*; utilization by *Bifidobacterium* and *Bacteroides*	EGFR, 5-HT1AR, nAChR, TCR, ERK/MAPK, PI3K/Akt, and G-protein-associated signaling pathways	Regulation of membrane integrity, cellular signaling, inflammation, immunity, autophagy, apoptosis, and cellular homeostasis
	Lipopolysaccharides	Gram-negative bacteria-derived lipopolysaccharides (LPS), including *Escherichia coli*, *Pseudomonas aeruginosa*, and *Bacteroides* spp.	Toll-like receptor-4 (TLR4) and Toll-like receptor-2 (TLR2) signaling pathways	Pro-inflammatory signaling, cytokine release, intestinal barrier dysfunction, and metabolic endotoxemia
Lipid mediators	Eicosanoids	Gut microbiota-modulated eicosanoids (PGs, TXs, LTs, and HETEs)	GPCR signaling, COX, LOX, and CYP450 pathways	Immune regulation, inflammatory responses, vascular tone regulation, maintenance of mucosal integrity, and promotion of inflammatory cascades
	Specialized pro-resolving mediators	Gut microbiota-associated SPMs (resolvins, lipoxins, protectins, and maresins)	GPCRs including LTB4R/BLT1, CMKLR1/ChemR23, ALX/FPR2, GPR32/DRV1, GPR18/DRV2, GPR37, LGR6, and GPR101	Resolution of inflammation, suppression of pro-inflammatory mediator production, neutrophil apoptosis, and maintenance of intestinal barrier integrity and tissue homeostasis
	Endocannabinoids and eCB-like lipids	Gut microbiota-associated Anandamide (AEA) and 2-arachidonoylglycerol (2-AG)	CB1, CB2, PPAR-α, TRPV1, GPR55, NF-κB, MAPK, and PI3K/Akt pathways	Regulation of energy homeostasis, inflammation, intestinal permeability, immune responses, cytokine synthesis, cell proliferation, and apoptosis

Schematic summary of dietary lipids, microbiota-derived lipid metabolites, membrane lipids, and lipid mediators together with their associated receptors/signaling pathways and biological effects in host–microbiota interactions. The figure was created based on the literature reviewed within the scope of this study.

PUFA, polyunsaturated fatty acid; n-3 LC PUFAs, omega-3 long-chain polyunsaturated fatty acids; SCFA, short-chain fatty acid; FFAR2 (GPR43), free fatty acid receptor 2; FFAR3 (GPR41), free fatty acid receptor 3; GPR109A (HCAR2), G protein-coupled receptor 109A/hydroxycarboxylic acid receptor 2; TGR5, Takeda G protein-coupled receptor 5; FXR, farnesoid X receptor; VDR, vitamin D receptor; PXR, pregnane X receptor; CAR, constitutive androstane receptor; HYA, 10-hydroxy-cis-12-octadecenoic acid; KetoC, 10-oxo-trans-11-octadecenoic acid; CLA, conjugated linoleic acid; MAPK, mitogen-activated protein kinase; MEK, MAPK/ERK, kinase; ERK, extracellular signal-regulated kinase; EGFR, epidermal growth factor receptor; 5-HT1AR, 5-hydroxytryptamine-1A receptor; nAChR, nicotinic acetylcholine receptor; TCR, T-cell receptor; PI3K/Akt, phosphoinositide 3-kinase/protein kinase B; GPCR, G protein-coupled receptor; LPS, lipopolysaccharide; TLR2, Toll-like receptor 2; TLR4, Toll-like receptor 4; PGs, prostaglandins; TXs, thromboxanes; LTs, leukotrienes; HETEs, hydroxyeicosatetraenoic acids; COX, cyclooxygenase; LOX, lipoxygenase; CYP450, cytochrome P450; SPMs, specialized pro-resolving mediators; LTB4R/BLT1, leukotriene B4 receptor 1; CMKLR1 (ChemR23/ERV1), chemokine-like receptor 1; ALX/FPR2, lipoxin A4 receptor/formyl peptide receptor 2; GPR32/DRV1, G protein-coupled receptor 32/D-resolvin receptor 1; GPR18/DRV2, G protein-coupled receptor 18/D-resolvin receptor 2; PD1, protectin D1; MaR1, maresin 1; AEA, anandamide; 2-AG, 2-arachidonoylglycerol; CB1, cannabinoid receptor type-1; CB2, cannabinoid receptor type-2; PPAR-α, peroxisome proliferator-activated receptor alpha; TRPV1, transient receptor potential vanilloid 1; NF-κB, nuclear factor-kappa B.

## 2 Methodology

This review was conducted to comprehensively evaluate the bidirectional interactions between gut microbiota and lipid mediators, particularly focusing on molecular signaling pathways, metabolic functions, and therapeutic implications in chronic diseases. Relevant studies published in English were identified through electronic database searches, including PubMed/MEDLINE, Scopus, and Web of Science.

The literature search was performed using the following keywords and Medical Subject Headings (MeSH) terms: “gut microbiota,” “intestinal microbiome,” “lipid mediators,” “short-chain fatty acids,” “bile acids,” “polyunsaturated fatty acids,” “specialized pro-resolving mediators,” “endocannabinoid system,” “sphingolipids,” “lipopolysaccharides,” “gut–liver axis,” “metabolic diseases,” “inflammation,” and “host–microbiota interaction.” Boolean operators (“AND”, “OR”) were applied to optimize the search strategy.

Original research articles, clinical studies, experimental animal studies, mechanistic studies, and relevant review articles analyzing microbiota-derived lipids, lipid-mediated signaling pathways, or interactions between dietary lipids and gut microbiota were included. Studies focusing on microbiota-unrelated pathways or lacking mechanistic and physiological relevance to lipid metabolism were excluded.

The collected evidence was narratively synthesized and categorized according to the origin and functional characteristics of the lipid-related molecules, including dietary lipids, microbiota-derived metabolites, structural membrane lipids, inflammatory lipid mediators, specialized pro-resolving mediators (SPMs), and endocannabinoid-related compounds.

This review aimed to provide an integrated conceptual framework summarizing the current knowledge on microbiota–lipid mediator interactions, while highlighting potential therapeutic approaches and areas requiring further mechanistic and clinical analysis.

## 3 Origin-based lipid categories and their interaction with microbiota

**FIGURE 1:**
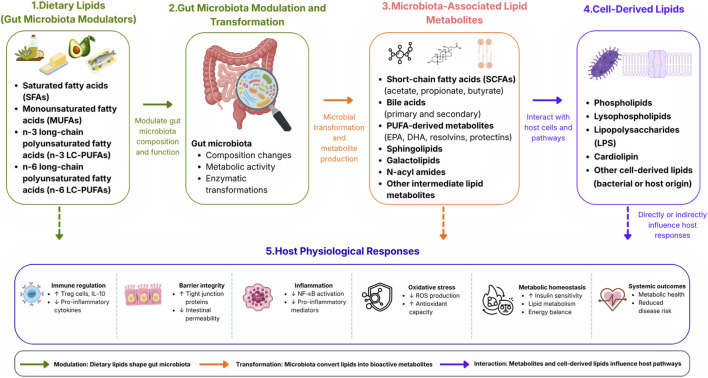
Classification and interactions of gut-associated lipids influencing the host’s physiological responses. Dietary lipids modulate the gut microbiota composition and function, leading to microbial transformation and production of microbiota-associated lipid metabolites. Together with cell-derived lipids, these metabolites influence the host’s physiological pathways associated with immune regulation, intestinal barrier integrity, inflammation, oxidative stress, and metabolic homeostasis. Created based on information synthesized from the literature.

Clarifying the relationship between the origin of gut lipids and their biological functions is essential for understanding microbiota–host interactions. In this context, distinguishing whether lipid profiles are derived from dietary sources or from cellular components, which are either bacterial or host in origin, represents a critical requirement, yet this distinction remains insufficiently defined in the current literature. Therefore, based on their origin and biological interactions, the lipids most commonly analyzed for their effects on host physiology can be broadly categorized into three major groups, namely, dietary lipids that modulate gut microbiota composition, microbiota-associated lipid metabolites generated through microbial transformation, and lipids originating from bacterial or host cellular components (Figure 1) (Carneiro et al., 2022).

### 3.1 Dietary lipids as modulators of gut microbiota composition

The gut microbiota profoundly shapes the processes of lipid absorption, digestion, metabolism, secretion, biotransformation, and detoxification, while dietary lipids, in turn, significantly influence the composition and metabolic capacity of the gut microbiota (Xiong, 2025; Schoeler et al., 2023; Lu et al., 2025). In general, the quantity of dietary fat modulates the microbial diversity; low-fat diets tend to increase microbial diversity, while high-fat diets reduce it, whereas the quality of dietary fat (i.e., fatty acid type and composition) is a critical determinant of microbial composition and metabolic output. As expected, high saturated fat intake promotes the dominance of detrimental microbial taxa, while diets rich in omega-3 PUFAs favor beneficial microbial communities (Mohammadi and Rudkowska, 2025). However, as this research field remains in its early stages, the specific amount of dietary fat required to support a healthy gut microbiota, the optimal levels of microbially derived lipids that benefit the host, and the extent to which these lipids are produced across different bacterial species have yet to be fully elucidated (Van Hul et al., 2024).

#### 3.1.1 n-3 LC PUFA and n-6 LC PUFA

The primary components of PUFAs, which are unsaturated groups with 2–6 double bonds in their structure, are α-linolenic acid (ALA), DHA, EPA from n-3 LC PUFAs, and linoleic acid (LA) from n-6 LC PUFAs (Saini and Keum, 2018). An essential component in controlling biological processes is the ratio of n-6 LC PUFAs, which have pro-inflammatory effects, to n-3 LC PUFAs, which have anti-inflammatory benefits (Fujisaka et al., 2018). Dietary n-3 and n-6 LC PUFAs are known to have different effects on the gut microbiota in humans (Mokkala et al., 2020). The gut microbiome is impacted by n-3 LC PUFAs in three ways. These include modifying the amount and makeup of the gut microbiota, regulating the amounts of SCFAs or their corresponding salts, and changing the presence of pro-inflammatory chemicals such as interleukin-17 (IL-17) and endotoxins (e.g., LPS). Although not selectively fermented substrates, omega-3 PUFAs can induce specific changes in the gut microbiota, shaping microbial communities, increasing microbial diversity, and promoting the growth of beneficial taxa. Therefore, due to their capacity to confer health benefits to the host through microbiota modulation, they are increasingly considered within the updated definition of prebiotics (Kumar et al., 2025; Zou et al., 2025).

Mechanistically, omega-3 PUFAs have been associated with increased abundance of beneficial bacteria such as *Bifidobacterium* and *Lactobacillus*, reduced levels of potentially pathogenic taxa such as Enterobacteriaceae, and restoration of the *Firmicutes*/*Bacteroidetes* ratio. These shifts are linked to strengthened gut barrier integrity and reduced intestinal inflammation (Kumar et al., 2025; Zou et al., 2025).

Clinical evidence, however, remains heterogeneous. In a randomized study in patients with type 2 diabetes (T2D), a 6-month intervention with sardine supplementation (100 g, 5 days per week) led to the abundance of *Bacteroides*-*Prevotella* and a reduced *Firmicutes/Bacteroidetes* ratio compared with that in controls (Balfegó et al., 2016). Similarly, in a 12-week double-blind randomized trial in hyper lipidemic individuals, supplementation with 4 g/day of plant-derived n-3 LC PUFAs improved intestinal dysbiosis by increasing the typical anaerobes, decreasing facultative anaerobes, and lowering the *Firmicutes/Bacteroidetes* ratio (Liu H. et al., 2022).

In contrast, in a multicenter randomized double-blind placebo-controlled trial involving 309 Chinese patients with T2D and hypertriglyceridemia, 12-week fish oil supplementation induced only modest alterations in the overall gut microbial diversity and taxonomic composition. Given that fish oil is predominantly absorbed in the small intestine and distributed systemically *via* the lymphatic and circulatory pathways, its direct interaction with the colonic microbiota may be limited, potentially explaining the modest microbiota-related effects observed (Lu et al., 2025).

The inconsistent findings regarding the effects of n-3 LC PUFAs on gut microbiota composition may be explained by several methodological and host-related factors. Variability in baseline dietary patterns, habitual fiber intake, ethnicity, sex distribution, age, metabolic health status, medication use, and physical activity levels may substantially influence microbiota responsiveness to dietary fat interventions (Asnicar et al., 2026). In addition, differences in the source of omega-3 fatty acids (marine *versus* plant-derived), dosage, intervention duration, and background dietary composition may contribute to the divergent microbial outcomes reported across studies (Zou et al., 2025). Lifestyle-related factors, including sleep quality and circadian rhythm disturbances, may also indirectly modulate the gut microbial composition and inflammatory responses, thereby influencing the observed effects of omega-3 supplementation on the microbiome (Sejbuk et al., 2024). Moreover, inter-individual variability in baseline microbiota composition may determine whether specific bacterial taxa respond favorably or remain unchanged following PUFA exposure (García-Mantrana et al., 2019).

Furthermore, a cross-sectional study in non-alcoholic fatty liver disease (NAFLD) patients reported no significant association between the dietary n-6/n-3 ratio and overall gut microbiota composition or disease severity (Heinzer et al., 2022), indicating that the ratio alone may not be a dominant determinant of microbial structure in humans.

Animal studies provide additional mechanistic insight. In mice, an ALA-rich diet significantly altered the jejunal microbiota composition, characterized by a decrease in *Firmicutes* and a concomitant increase in *Bacteroidetes* (Todorov et al., 2020). Likewise, an 8-week high-fat diet enriched with EPA and DHA (1 g per 100 g diet) increased the *Bifidobacterium (Actinobacteria)* levels while decreasing the *Firmicutes, Tenericutes, and* Enterobacteriaceae *(Proteobacteria)* groups (Robertson et al., 2017).

Beyond their role in modulating the gut bacterial composition, n-3 LC PUFAs also act as substrates for the synthesis of bacterial metabolites such as SCFAs (Kumar et al., 2025; Zou et al., 2025). Supplementation with n-3 PUFAs has been associated with increased luminal SCFA levels and a transient rise in SCFA-producing bacteria, including *Bifidobacterium*, *Roseburia*, and *Lactobacillus* (Kumar et al., 2025; Watson et al., 2018). Notably, both dietary intake and supplementation of n-3 LC PUFAs have been shown to alter the gut microbial composition by increasing butyrate-producing members of the *Lachnospiraceae* family, which are associated with the anti-inflammatory SCFA butyrate, while reducing the abundance of *Faecalibacterium* in certain contexts (Costantini et al., 2017). In addition, elevated tissue levels of omega-3 fatty acids have been shown to enhance the production of intestinal alkaline phosphatase (IAP), thereby modulating the gut ecosystem. This IAP-mediated regulation leads to reduced LPS production and improved intestinal barrier function, ultimately attenuating metabolic endotoxemia and the inflammatory response (Kaliannan et al., 2015).

Diets rich in n-6 fatty acids are said to have a pro-inflammatory effect on the colon, increasing the risk of colon cancer and obesity (Selmin et al., 2021). Bacteria with pro-inflammatory characteristics, such as *Clostridium* spp., segmented filamentous bacteria, and *Clostridium cluster XI*, have been found to be more prevalent in diets high in LA (Monguchi et al., 2017). In line with this, another study showed that diets enriched with n-6 LC PUFAs led to an increase in Enterobacteriaceae levels (Ghosh et al., 2013). It is stated that the increase in these Gram-negative bacteria raises serum LPS levels, which increase intestinal permeability and contribute to the development of inflammation and metabolic diseases (Salguero et al., 2019). Furthermore, another study discovered that consuming large amounts of LA changed the composition of the gut microbiota and decreased the abundance of *Sutterella* spp. and *Akkermansia* spp. in rats (Shrestha et al., 2020). In mice given a diet rich in fat and LA, where 45% of energy comes from fat, an increase in the quantity of the Desulfovibrionaceae *(Proteobacteria)* and Clostridiaceae *(Firmicutes)* families was shown, while a significant declines were noted in the Prevotellaceae*,* Bacteroidaceae*,* and Rikenellaceae *(Bacteroidetes)* families (Hildebrandt et al., 2009).

Omega-3 and omega-6 PUFAs modulate the common signaling pathways, including nuclear factor-kappa B (NF-κB), NOD-like receptor family pyrin domain containing 3 (NLRP3) inflammasome, mitogen-activated protein kinase (MAPK; extracellular signal-regulated kinase [ERK] and c-Jun N-terminal kinase [JNK]), epidermal growth factor receptor (EGFR)/Raf-1, and G protein-coupled receptor 120 (GPR120), although differences in signaling kinetics and activation intensity have been reported. Through the regulation of these pathways, both omega-3 and omega-6 PUFAs influence inflammatory responses, intestinal barrier integrity, and cellular homeostasis in intestinal epithelial cells (Kumar et al., 2025; Mobraten et al., 2013).

On the other hand, the metabolism of dietary PUFAs by the gut microbiota leads to the generation of bioactive lipid derivatives (Zinkow et al., 2025). Certain bacterial taxa, such as *Bacillus proteus* and *Lactobacillus plantarum*, participate in the transformation of omega-3 and omega-6 fatty acid precursors, including ALA and LA, leading to the production of conjugated fatty acid forms such as conjugated linoleic acid (CLA) and conjugated α-linolenic acid (CALA) (Kumar et al., 2025). Additionally, *Lactobacillus plantarum* has the capacity to hydroxylate the double bonds of PUFAs (e.g., LA) and subsequently oxidize the resulting hydroxyl groups to ketones. As a result of these reactions, multiple bioactive intermediates are produced, including 10-hydroxy-cis-12-octadecenoic acid (HYA). This compound is linked to favorable metabolic outcomes and anti-inflammatory effects in the host (Matsushita et al., 2025).

#### 3.1.2 Monounsaturated fatty acids

Monounsaturated fatty acids (MUFAs) are a class of unsaturated fatty acids that contain one cis double bond in their hydrocarbon backbone that can be found in a variety of plant sources, such as avocados, seeds, nuts, and olive oil (Öz et al., 2022). The effects of a MUFAs-rich diet on the gut flora have been inconsistently reported. One study indicated that gut microbiota abundance and diversity was not impacted by MUFAs, or that increased *Bifidobacterium* spp. abundance might even be negatively correlated with MUFAs intake (De Wit et al., 2012). Similarly, a different study discovered that phylum distribution, diversity indices, and the ratio of *Bacteroidetes* to *Firmicutes* were unaffected by high-MUFAs diets (Wolters et al., 2019).

There are also studies showing that MUFAs increase beneficial bacteria. In a 4-week clinical trial, MUFAs-rich oils (canola, canola oleic oil, and canola/DHA) were found to markedly change the gut microbiota composition in individuals at the risk of metabolic syndrome (MetS). Consumption of these oils increased the abundance of various beneficial bacterial genera within the *Firmicutes* and *Bacteroidetes* phyla, while potentially harmful *Isobaculum* levels decreased; conversely, an increase in Enterobacteriaceae levels was observed (Pu et al., 2016). A study in mice found that long-chain MUFAs treatment altered the gut microbiota composition, leading to a decrease in the *Firmicutes/Bacteroidetes* ratio and an increase in *Akkermansia*, while also increasing SCFAs production (Tsutsumi et al., 2021). In addition, an SFA-rich diet has been shown to markedly alter the microbiome community structure, whereas a MUFA-rich diet partially reversed these changes. In this context, the SFA group exhibited increased abundances of *Bacteroides*, *Dubosiella*, and *Turicibacter*, along with a decrease in *Lactobacillus*, while the MUFA diet partially normalized these alterations (Noureldein et al., 2025). Moreover, an experimental study analyzing the effects of oleic acid-rich oils on gut microecology demonstrated that high-oleic peanut oil (HOPO) effectively ameliorated gut microbial dysbiosis induced by a high-fat and high-fructose diet. HOPO supplementation was associated with a reduction in pathogenic fungal taxa (*Aspergillus*, *Penicillium*, and *Candida*) and an improvement in the fungal-to-bacterial diversity ratio. Moreover, normalization of serum metabolites related to bile acid metabolism and coenzyme A biosynthesis indicates that oleic acid-rich fats may exert regulatory effects not only on bacterial communities but also on the gut mycobiome and host metabolic pathways (Zhao Z. et al., 2025).

In a study of 20 obese men with coronary heart disease, the participants were randomly allocated to either a Mediterranean diet (35% fat and 22% MUFAs) or a low-carbohydrate and high-cholesterol diet (28% fat and 12% MUFAs). When comparing to the baseline, the MUFAs-rich Mediterranean diet increased the abundance of the species *Parabacteroides distasonis* and the genera *Roseburia* and *Oscillospira* while decreasing the genus *Prevotella* (Haro et al., 2016).

Conversely, some studies have found that MUFAs have negative effects on the microbiota (Wolters et al., 2019; Röytiö et al., 2017; Pu et al., 2013). High intake of MUFAs was found to be inversely correlated with microbial richness and diversity in a cross-sectional study (Röytiö et al., 2017). Furthermore, diets rich in MUFAs have been reported to show a positive correlation with pathogenic bacteria of the Enterobacteriaceae family (Wolters et al., 2019; Pu et al., 2013).

These inconsistent results may be associated with differences in the dietary context and the participants’ characteristics between studies. MUFAs are usually consumed as part of a complex dietary pattern, such as the Mediterranean diet, which is also rich in fiber, polyphenols, vegetables, and fermented foods. Thus, it might be challenging to determine the independent effect of MUFAs on gut microbiota composition (Nagpal et al., 2019). Moreover, inter-study variability might also be explained by sex-specific hormonal profiles, ethnic and geographic differences in habitual diet, sleep behavior, physical activity level, obesity status, and metabolic disease burden (Hasan and Yang, 2019). Variations in the intervention duration, techniques used for microbial analysis, and initial microbiota composition may also play a role in the mixed findings reported between MUFA intake and microbial diversity (Aslam et al., 2026).

Heatmap findings derived from recent review studies indicate that MUFA intake induces taxon-specific alterations in the gut microbiota. MUFA consumption has been associated with increased abundance of SCFA-related beneficial taxa, including *Faecalibacterium*, *Fusibacter*, and members of *Actinobacteria*, while a decrease in *Lactobacillus* and a tendency toward increased abundance of certain members of the *Firmicutes* phylum have also been observed. This compositional pattern indicates that MUFAs modulate the gut microbiota in a selective and context-dependent manner rather than exerting a uniform effect. Nevertheless, when the overall microbial structure is considered, MUFAs appear to be associated with a more balanced and metabolically favorable gut microbial profile (Mohammadi and Rudkowska, 2025).

#### 3.1.3 Saturated fatty acids

Saturated fatty acids (SFAs) lack double bonds between carbon atoms, which makes them solid at room temperature (Agregán et al., 2022). SFAs, which are present in foods such as red meat, cheese, butter, and coconut oil, are reported to be associated with metabolic diseases and disruption of the gut microbiota balance (Mozaffarian and Ludwig, 2015). One study found that SFAs intake had a greater impact on the gut microbiota than MUFAs or PUFAs intake (Schoeler et al., 2023). Higher SFAs intake is reported to be associated with decreased microbial richness and diversity in humans, potentially triggering systemic inflammation (Wolters et al., 2019; de Queiroz Cavalcanti et al., 2023).

A dietary pattern characterized by high intake of SFAs and low consumption of fiber and PUFAs induces characteristic shifts in gut microbiota composition. Under this nutritional profile, decreased abundances of *Bacteroidetes*, *Prevotella*, and *Bifidobacterium* have been observed, alongside an increased tendency in *Lactobacillus* and particularly within the *Firmicutes* phylum. Additionally, elevations in taxa such as *Ralstonia*, members of the Enterobacteriaceae family, *Helicobacter pylori*, and *Akkermansia* have been reported. These compositional alterations indicate that high SFA and low fiber/PUFA intake may shift the gut ecosystem toward a dysbiotic state, potentially fostering a microbial environment that is conducive to inflammatory processes (de Queiroz Cavalcanti et al., 2023; Cândido et al., 2018). One study indicated that SFAs are positively correlated with *Fusobacterium*, which is identified as an opportunistic pathogen (Xu et al., 2022).

Low-fiber diets high in fat and sugar appear to stimulate the proliferation of microbial groups that use SFAs for energy. Such diets can trigger inflammatory responses by increasing the production of metabolites associated with weakening of the intestinal barrier (Aho et al., 2021). Furthermore, it has been stated that increased SFAs intake raises the levels of Gram-negative bacteria-specific LPS, which in turn stimulates cytokine production and increases nitric oxide production, which contributes to inflammation and oxidative stress in the ileum (TL et al., 2018).

A summary of clinical and preclinical studies analyzing the effects of dietary fatty acids on the gut microbiota composition is presented in Table 2.

**TABLE 2:** Summary of the clinical and preclinical studies evaluating the effects of dietary lipids on gut microbiota.

Study type	Lipid type	Population/Model	Intervention	Main gut microbiota findings	References
Clinical	n-3 LC PUFA	Patients with T2D	Sardine-enriched diet	↑ *Bacteroides-Prevotella*, ↓ *Firmicutes/Bacteroidetes* ratio	Balfegó et al. (2016)
Clinical	n-3 LC PUFA	Hyperlipidemic adults	Plant-derived n-3 PUFA supplementation	↑ Anaerobic bacteria, ↓ *Firmicutes/Bacteroidetes* ratio	Liu et al. (2022b)
Clinical	n-3 LC PUFA	Patients with T2D and hypertriglyceridemia	Fish oil supplementation	Modest alterations in microbial diversity and composition	Lu et al. (2025)
Clinical	n-6/n-3 ratio	Patients with NAFLD	Cross-sectional dietary analysis	No significant association with microbiota composition or disease severity	Heinzer et al. (2022)
Preclinical	ALA-rich diet	Mice	ALA-rich diet	↓ *Firmicutes*, ↑ *Bacteroidetes*	Todorov et al. (2020)
Preclinical	EPA/DHA	Mice	Omega-3 PUFA intervention	↑ *Bifidobacterium*, ↓ Enterobacteriaceae	Robertson et al. (2017)
Preclinical	n-6 LC PUFA (LA)	Rats	High-LA diet	↓ *Sutterella* spp., ↓ *Akkermansia* spp.	Shrestha et al. (2020)
Preclinical	n-6 LC PUFA (LA)	Mice	LA-rich high-fat diet	↑ Desulfovibrionaceae and Clostridiaceae, ↓ Bacteroidaceae	Hildebrandt et al. (2009)
Clinical	MUFA	Adults at risk of MetS	Canola oil-based intervention	Altered *Firmicutes*- and *Bacteroidetes*-related taxa; ↓ *Isobaculum*	Pu et al. (2016)
Clinical	MUFA	Obese men with CHD	Mediterranean diet rich in MUFAs	↑ *Roseburia* and *Oscillospira*, ↓ *Prevotella*	Haro et al. (2016)
Clinical	MUFA	Cross-sectional cohort	High MUFA intake	↓ Microbial richness and diversity	Röytiö et al. (2017)
Preclinical	MUFA	Mice	Long-chain MUFA treatment	↑ *Akkermansia*, ↑ SCFA production	Tsutsumi et al. (2021)
Preclinical	MUFA	Rats	High-oleic peanut oil supplementation	Modulated gut mycobiome and improved dysbiosis-related profile	Zhao et al. (2025a)
Clinical/Preclinical	SFA	Human and animal evidence	High-SFA dietary patterns	Associated with reduced microbial diversity and pro-inflammatory microbiota alterations	de Queiroz Cavalcanti et al. (2023)

ALA, alpha-linolenic acid; CHD, coronary heart disease; DHA, docosahexaenoic acid; EPA, eicosapentaenoic acid; LA, linoleic acid; LC PUFA, long-chain polyunsaturated fatty acid; MetS, metabolic syndrome; MUFA, monounsaturated fatty acid; NAFLD, non-alcoholic fatty liver disease; SCFA, short-chain fatty acid; T2D, type 2 diabetes.

### 3.2 Microbiota-derived lipid metabolites

#### 3.2.1 Short-chain fatty acids

The three SCFAs that gut bacteria create are butyrate, propionate, and acetate. These SCFAs have positive impacts on energy metabolism and protect the host through a variety of local anti-inflammatory activities (Cundra et al., 2022). Several bacterial species, such as *Lactobacillus, Bifidobacterium, Prevotella,* and *Bacteroides*, ferment fibers to create SCFAs (Mann et al., 2024; Markowiak-Kopeć and Śliżewska, 2020). Both butyrate and propionate have low systemic concentrations (20% and 20%), while acetate levels are higher (60%) (Wisniewski et al., 2019).

Acetate, a two-carbon SCFA, is a vital source of energy for host cells, especially in peripheral organs and the brain, and it exhibits anti-inflammatory properties. It protects the intestinal epithelium by reducing intestinal permeability. Butyrate, a four-carbon SCFA, is the primary energy source for colonocytes, possesses strong anti-inflammatory effects, and plays a role in regulating intestinal permeability. Propionate is a three-carbon SCFA that is mostly broken down in the liver and affects the release of intestinal hormones such as glucagon-like peptide-1 (GLP-1) and peptide YY (Zhang et al., 2024; Mansuy-Aubert and Ravussin, 2023).

SCFAs have several positive effects on human health. They serve an important role in many physiological functions, including maintaining the intestinal barrier integrity, regulating neurological function, metabolic balance, and modulating immune response. Butyrate strengthens the intestinal epithelial integrity through increased tight junction protein and Mucin-2 expression, while also exhibiting oxidative stress-reducing and anticancer effects. SCFAs also influence the nervous system, regulating intestinal gluconeogenesis, neuroinflammation, and the expression of neurotrophic factors, thus being linked to mood and cognitive function. Metabolically, acetate, propionate, and butyrate play a role in appetite control, regulation of glucose and lipid metabolism, and weight management. Furthermore, SCFAs are reported to have positive effects on cardiometabolic health and are associated with reductions in blood pressure and prothrombotic factors. Finally, SCFAs suppress inflammation by modulating both innate and acquired immune responses, and butyrate, in particular, reduces intestinal inflammation through an increase in anti-inflammatory cytokines (Mansuy-Aubert and Ravussin, 2023; Fusco et al., 2023).

Diet is an important element that controls and regulates the makeup and function of an individual’s gut microbiota (Rinninella et al., 2019). Microorganisms in the colon consume fermentable fibers as substrates and transform them into a variety of metabolites, mostly SCFAs. Probiotics, prebiotic fiber supplements, and dietary fiber all influence the gut microbiota and typically boost the production of SCFAs (Cronin et al., 2021).

#### 3.2.2 Bile acids

Bile acids (BAs) are generated in hepatocytes *via* the enzymatic transformation of cholesterol into amphipathic molecules that facilitate lipid solubilization within the intestinal lumen, thereby supporting the absorption of dietary lipids and fat-soluble vitamins (Di Ciaula et al., 2018). Following food intake, bile is released into the duodenum; approximately 95% of the secreted bile acids are reabsorbed in the jejunum and ileum and returned to the liver *via* the portal vein through enterohepatic circulation, while approximately 5% are excreted in the feces, and nearly 15% of the primary bile acids that escape absorption in the terminal ileum reach the colon and remain within the intestinal environment (Guzior and Quinn, 2021). This residual pool undergoes bacterial modification, leading to the formation of secondary BAs that are primarily mediated by resident genera such as *Bacteroides* and *Clostridium* (Fang et al., 2024; Mohammadi and Rudkowska, 2025).

This biotransformation occurs through three major enzymatic steps: (1) deconjugation mediated by bacterial bile salt hydrolases (BSHs), (2) 7α-dehydroxylation through sequential enzymatic reactions, and (3) epimerization *via* hydroxysteroid dehydrogenases (Fleishman and Kumar, 2024; Chiang, 2013; Gérard, 2013). In humans, the primary BAs cholic acid (CA) and chenodeoxycholic acid (CDCA) are converted to the secondary BAs deoxycholic acid (DCA) and lithocholic acid (LCA), respectively, through the removal of the C7 hydroxyl group (Li et al., 2024a). The secondary BA pool is quite diverse, with DCA and LCA being the predominant components (Guzior and Quinn, 2021).

The generation of secondary BAs alters both their chemical structure and receptor-binding properties, thereby modifying the biological activity (Hang et al., 2019). These molecules function as potent signaling mediators through the nuclear Farnesoid X receptor (FXR) and the Takeda G protein-coupled receptor 5 (TGR5); by acting as agonists at these receptors, they trigger transcriptional and metabolic responses in immune cells, hepatocytes, and intestinal epithelial cells, thereby contributing to the coordinated regulation of glucose and lipid metabolism, energy expenditure, and inflammation (Lu Q. et al., 2024; Baars et al., 2015). As evident, beyond their digestive functions, bile acids also act as amphipathic signaling molecules involved in glucose metabolism and immune regulation (Zhao et al., 2022).

**FIGURE 2:**
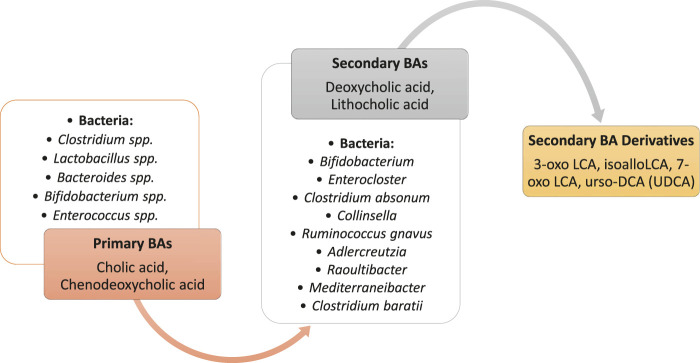
Dehydroxylation of bile acids (Wang J. et al., 2023; Su et al., 2023) (BA: Bile acid, LCA: Lithocholic Acid, 3-oxo LCA: 3-Oxolithocholic Acid, isoalloLCA: Isoallolithocholic Acid, 7-oxo LCA: 7-Oxolithocholic Acid, UDCA: Ursodeoxycholic Acid).

BAs not only shape the composition of the gut microbiota but are also enzymatically transformed by microbial communities into novel bile acid derivatives and signaling molecules (Fuchs et al., 2025). In this process, bacterial BSH enzymes mediate the deconjugation of conjugated primary bile acids in the small intestine with glycine, taurine, and other amino acids (Figure 2) (Wang J. et al., 2023; Su et al., 2023). Conjugated primary BAs are deconjugated in the distal portion of the small intestine by anaerobic bacteria, including species such as *Bacteroides, Clostridium, Lactobacillus*, and *Bifidobacterium* (Fiorucci et al., 2018). Later, subsequent modifications, including oxidation and epimerization of hydroxyl groups, are performed by genera such as *Bacteroides, Escherichia, Eggerthella, Eubacterium, Clostridium*, and *Peptostreptococcus* (Lamichhane et al., 2021). While certain hydrophobic BAs may disrupt cellular membranes, others protect the intestinal epithelium against pathogens such as *Clostridioides difficile* (Hang et al., 2019).

In addition to their signaling roles, secondary BAs contribute to the regulation of host metabolic pathways and innate immune responses (Ramírez-Pérez et al., 2018). By interacting with the receptors they express, primary and secondary BAs and their derivatives regulate the development and activity of macrophages, dendritic cells (DCs), myeloid-derived suppressor cells (MDSC), regulatory T cells (Treg), B cells, T helper 17 (Th17) cells, natural killer T (NKT) cells, innate lymphoid cells (ILCs), CD4 cells, and CD8 cells (Su et al., 2023; Campbell et al., 2020). Numerous diseases can be caused by the disruption of the complex interactions between BAs and the microbiota. Immune-related problems encompass intestinal disorders, metabolic diseases, malignancies, neurological diseases, allergies, autoimmune disorders, and infectious diseases, all of which are associated with the breakdown of homeostasis (Grüner and Mattner, 2021).

#### 3.2.3 PUFA-derived intermediate metabolites

PUFA-derived intermediate metabolites generated by the gut microbiota also function as significant metabolic regulators, and dietary PUFAs appear to have a regulatory role in microbiota and cell communication (Kishino et al., 2013). The gut microbiota converts dietary PUFAs into LA and its α-LA derivatives, which include hydroxy fatty acids, conjugated fatty acids, oxo fatty acids, and partly saturated trans-fatty acids (Carneiro et al., 2022).

These metabolites include HYA, 10-hydroxy-octadecanoic acid (HYB), 10-hydroxy-trans-11-octadekenoic acid (HYC), 10-oxo-cis-12-octadekenoic acid (KetoA), 10-oxo-octadecanoic acid (KetoB), and 10-oxo-trans-11-octadekenoic acid (KetoC) (Miyamoto et al., 2019). A study indicates that the most prominent gut bacteria PUFAs metabolite affecting host metabolism is HYA. Gut bacteria has been found to reduce inflammation by turning excess dietary LA into HYA (Miyamoto et al., 2019). It has also been reported that HYA suppresses the growth of *Helicobacter pylori* and *Helicobacter suis*, and thus, these PUFAs metabolites exert a protective effect by preventing the colonization of pathogens (Matsui et al., 2017). Another study found that *via* inhibiting Ras/Raf/MAPK (MEK)/extracellular-signal-regulated kinase (ERK) phosphorylation, HYA and KetoC have anti-inflammatory actions in mice microglial cells (Ikeguchi et al., 2018).

Among PUFA-derived metabolites, CLA has gained particular attention due to its biological activity. CLA, a form of LA containing conjugated double bonds, occurs naturally in ruminant-derived foods. Additionally, *Butyrivibrio fibrisolvens*, *Lactococcus lactis, Propionibaterium freudenrehichii*, and some *Streptococcus*, *Bifidobacterium*, and *Limosilactobacillus* species can also produce CLA. CLA has many functions, including preventing breast and colon cancers, skin tumors, and diabetes; reducing body fat accumulation and atherosclerosis; and modulating the immune system (Saika et al., 2019; Badawy et al., 2023).

#### 3.2.4 N-acyl amides

N-acyl amides, also known as lipoamines, are signaling molecules formed by the binding of saturated, unsaturated, or hydroxylated fatty acids to amines including dopamine, ethanolamine, or amino acids, and they have a function in cellular communication (Prakash and Kamlekar, 2021). These compounds play a role in fundamental biological reactions including immunological homeostasis, determination of adipose tissue volume, and the regulation of energy expenditure associated with obesity, while also contributing to pain perception, memory functions, and insulin level control (Mannochio-Russo et al., 2025).

Recent investigations have revealed that the human gut microbiota is not limited to producing metabolic byproducts but can also synthesize biologically active lipid signals that are structurally and functionally similar to the host’s endocannabinoid system (ECS) (Roussel et al., 2024; Castonguay-Paradis et al., 2023). These microbial lipids are primarily N-acylamides (e.g., N-acylethanolamines) and some monoacylglycerols, which exhibit significant structural and functional similarities to the host endocannabinoids (Silvestri and Di Marzo, 2023; Hosseinkhani et al., 2021).

Microbial-derived N-acyl amides and monoacylglycerols (e.g., oleoylethanolamide and 2-oleoylglycerol) can modulate metabolic processes by regulating enterohormonal responses and stimulating GLP-1 release. These lipids are also reported to have effects on signaling networks associated with immunity and inflammation. In this context, it appears that microbiota-produced N-acyl amides establish a direct molecular communication mechanism between microbiota and the host by targeting the host ECS signaling and play a decisive role in gastrointestinal homeostasis, immune tolerance, and metabolic balance (Pires et al., 2024; Silvestri and Di Marzo, 2023).

### 3.3 Cell-component-derived structural lipids

#### 3.3.1 Membrane lipids

Membrane lipids synthesized by gut microbes, such as phospholipids, sphingolipids, sulfonolipids, and prenol lipids, also function as signaling molecules in the host (Brown et al., 2023). Phospholipids represent the most abundant and structurally diverse components of bacterial membranes. Phosphatidylethanolamine is the most prevalent phospholipid produced by the gut microbiota (Sohlenkamp and Geiger, 2016). Bacteria can modify the fatty acid chain length, degree of branching, and saturation level of phospholipids in order to adapt to the environmental conditions (De Carvalho and Caramujo, 2018). However, the interactions between commensal bacterial phospholipids and host cells are not yet fully understood (Brown et al., 2023; Ryan et al., 2023; Boldyreva et al., 2021).

Aside from being structural elements of cell membranes, phospholipids play an active role in numerous essential cellular processes, including the regulation of membrane trafficking, localization and functional control of membrane proteins, autophagy, cellular signal transduction, cell proliferation and differentiation, cell migration, and apoptosis (Frydrych et al., 2025). Due to these multifaceted functions, the balance of phospholipid metabolism is critical for cellular homeostasis. Numerous conditions, such as dyslipidemia, atherosclerosis, liver diseases, respiratory disorders, autoimmune diseases, neurological disorders, cardiovascular and skeletal muscle diseases, and malignancies, are brought on by impaired phospholipid metabolism (Morita and Ikeda, 2022; Yoon et al., 2021).

Glycerophospholipids and sphingomyelins are the two primary types of phospholipids. Each type of lipid has different structural and functional properties that enable them to fulfill specific roles in the cell membrane (Ventura et al., 2022). Glycerophospholipids consist of a glycerol backbone, two fatty acids, a phosphate group, and a head group, which gives the phospholipid its name. Diacylglycerol is formed by the esterification of the hydroxyl groups at the sn-1 and sn-2 positions of glycerol with fatty acids, while phosphatidic acid is formed by adding a phosphate group to the sn-3 position (Osawa et al., 2024). Phosphatidic acid is the simplest phospholipid and serves as a fundamental building block in the synthesis of other phospholipids. The addition of different head groups to the phosphate of phosphatidic acid creates various phospholipids. Phosphatidylcholine, formed by the attachment of choline to one of these head groups, is the most common phospholipid in cell membranes and plays an important role in maintaining the membrane structure and function (Osawa et al., 2024; Cockcroft, 2021; Van Meer et al., 2008).

Plasmalogens are glycerophospholipids, with the naturally occurring types being phosphatidyl choline and phosphatidylethanolamine. Plasmalogens have a protective effect against oxidative stress and are found in high concentrations in neurological tissues (Paul et al., 2019). Because some bacteria in the gut microbiota can synthesize plasmalogens, it is thought that these lipids may contribute to host metabolism (Brown et al., 2023; Ryan et al., 2023).

Sphingolipids, which are long-chain lipids with amide-linked fatty acyl chains, play an important part in the widespread structural makeup of cell membranes, specifically in the neurological system (Quinville et al., 2021). These molecules can be obtained from the diet or synthesized by commensal gut microbes. The majority of the phylum *Bacteroidetes* (including genera such as *Bacteroides, Porphyromonas*, *Prevotella, Parabacteroides,* and *Flectobacillus*) and a few members of the phylum *Chlorobi* (including *Chlorobium*) are known to produce sphingolipids (Olsen and Jantzen, 2001). In addition, dietary sphingolipids are reported to be utilized by bacterial groups such as *Bifidobacterium* and *Bacteroides* (Lamichhane et al., 2021). This indicates that bioactive lipids that can access the gut microbiome influence the host’s microbial composition (Lee et al., 2021). Although eukaryotes are the most prevalent producers of sphingolipids, bacteria can also generate them. Nonetheless, bacterial and eukaryotic sphingolipids differ significantly. Bacterial sphingolipids typically feature single-chain length sphingoid backbones and fatty acyl chains with methylation or hydroxylated structures. In contrast, mammals primarily manufacture double-chained and linear sphingoid backbones (Heaver et al., 2018).

Mammalian sphingolipids are important signaling molecules that govern several cellular processes in the host, including cellular differentiation, inflammation, immunity, autophagy, apoptosis, and cell proliferation (Quinville et al., 2021; Hannun and Obeid, 2018). Disruptions in sphingolipid homeostasis cause a variety of ailments, including neurological, cancer, metabolic, cardiovascular, inflammatory, and infectious diseases (Jamjoum et al., 2024). The potential of bacterial sphingolipids and their interaction with the host are still not fully understood. Given that mammalian sphingolipids play key roles in host biology, it is assumed that bacterial sphingolipids alter host phenotypes *via* pathways spanning from proliferation to insulin signaling. Bacterial sphingolipids have been found to migrate from epithelial cells into internal organs, changing the host’s metabolism (Bai et al., 2023; Le et al., 2022).

Sulfonolipids, which are structurally related to phosphoceramides, are primarily produced by bacteria belonging to the *Bacteroidetes* phylum. Certain sulfonolipids activate toll-like receptor-4 (TLR-4) signaling and induce strong pro-inflammatory responses in macrophages (Walker et al., 2017). Increased dietary fat intake has also been associated with enhanced sulfonolipid production and TLR-4 linked intestinal inflammation (Kim et al., 2012).

Membrane lipids actively participate in cellular signal transduction by modulating the activity of multiple receptors, including epidermal growth factor receptor (EGFR), 5-hydroxytryptamine-1A receptor (5-HT1AR), nicotinic acetylcholine receptor (nAChR), μ-opioid receptor, and T-cell receptor (TCR). Lipids such as cholesterol, sphingolipids, gangliosides, and ceramide regulate extracellular signal-regulated kinase/mitogen-activated protein kinase (ERK/MAPK), phosphoinositide 3-kinase/Akt (PI3K/Akt), and G-protein-associated signaling pathways, thereby influencing receptor clustering, ligand binding, channel opening, inflammation, and immune responses. Furthermore, alterations in membrane lipid composition can activate or suppress downstream signaling pathways, thereby shaping cellular homeostasis (Arish et al., 2015; Sunshine and Iruela-Arispe, 2017).

#### 3.3.2 Lysophospholipids

Lysophospholipids, also called hydrolyzed lipids, are lipids that contain a single alkyl or acyl chain in their structure. These molecules are formed when a phospholipid loses one of its fatty acid chains through hydrolysis (Heringdorf, 2008). Based on their backbone structure, lysophospholipids are divided into two main groups, namely, lysoglycerophospholipids and lysophingolipids. Both groups are amphipathic molecules, having a hydrophilic head group connected to a glycerol or sphingosine backbone containing a lengthy hydrophobic carbon chain. Due to these structural characteristics, lysophospholipids exhibit different physicochemical and biological properties than their parent phospholipid or sphingolipid forms (Farooqui and Horrocks, 2007).

Intracellular lysophospholipids serve as intermediates in the manufacture of other complex lipids; hence, their quantities are typically low. In contrast, they are found in very significant levels in extracellular settings such as plasma and interstitial fluid, where they are primarily conveyed bound to specialized protein carriers. High concentrations of lysophospholipids have been reported to disrupt the integrity of the cell membrane, leading to membrane destabilization and cell lysis (Xu et al., 2001). They are known to perform a wide range of tasks, such as controlling cellular differentiation, growth, proliferation, migration, and apoptosis (Ali and Szabó, 2023).

#### 3.3.3 Lipopolysaccharides

LPS is a structural component of Gram-negative bacteria, consisting of long carbohydrate chains covalently linked to lipids (Wassenaar and Zimmermann, 2018). LPS is considered a component recognized by the innate immune system that signals the presence of a pathogen. As a result, LPS is frequently characterized as a factor that significantly boosts the host immune response and causes the release of cytokines that promote inflammation (Farhana and Khan, 2021). However, the strength and nature of the host’s reaction varies depending on the pathogenic or commensal qualities of the bacterium that produces the LPS, along with the structural properties of the LPS itself. For instance, LPS produced from *Escherichia coli* has been reported to lead to significantly higher increases in IL-1β, IL-6, IL-8, and tumor necrosis factor-α levels than LPS derived from *Pseudomonas aeruginosa*. Furthermore, *E. coli* LPS has been shown to more strongly stimulate monocyte chemo-attractant protein-1 (MCP-1) production *via* TLR-4 (Cho et al., 2014). LPS is known to stimulate pro-inflammatory responses *via* TLR-4 (Kwiatkowska and Ciesielska, 2018). However, LPS isolated from *Bacteroides* species of the microbiota are reported to be less inflammatory and generate different signaling profiles *via* both TLR-2 and TLR-4 (Hug et al., 2018).

Diet can modulate both the structure and circulating levels of LPS in the host by influencing the abundance of pathogenic bacteria and the integrity of the intestinal epithelial barrier (Wan et al., 2019). In particular, Western-style dietary patterns characterized by high fat and protein intake and low fiber content have been shown to disrupt the gut microbiota composition and compromise the intestinal barrier function This facilitates increased passage of LPS from the intestinal lumen into the host circulation and promotes LPS translocation *via* intracellular or extracellular mechanisms (Stephens and von der Weid, 2020; Mohr et al., 2022).

#### 3.3.4 Cardiolipin

The primary components of mitochondrial membranes are phospholipids. A distinct phospholipid found and produced in the inner mitochondrial membrane, cardiolipin (CL), makes up 15%–20% of all mitochondrial phospholipids (Thatavarthy et al., 2025). They are mostly located in the inner membrane of bacterial cells and the mitochondria of eukaryotic cells (Lin and Weibel, 2016). By preserving the appropriate structure and shape of mitochondrial membranes and controlling the activity of numerous proteins and enzymes involved in mitochondrial function, CL plays a crucial part in mitochondrial metabolism (Paradies et al., 2019).

CL deficiency can lead to the disruption of the structural integrity of mitochondrial respiratory supercomplexes and, consequently, a decrease in energy production. Therefore, CL is considered a fundamental structural component that maintains the stability of supercomplexes by holding respiratory chain complexes together. Supercomplex disintegration increases the generation of reactive oxygen species (ROS) in the mitochondria, which promotes oxidative damage to CL (Zegallai and Hatch, 2021).

The high sensitivity of CL’s molecular structure to lipid peroxidation leads to peroxidized CL triggering cytochrome c release, initiating the apoptotic process. Furthermore, the localization of CL in the inner mitochondrial membrane, where ROS production is concentrated, makes this lipid even more vulnerable to oxidative damage (Ye et al., 2016). An imbalance between ROS formation in mitochondria and antioxidant defense systems such as superoxide dismutase increases mitochondrial dysfunction, thus exacerbating cellular damage (Ray et al., 2012).

## 4 Lipid mediators: biosynthesis, activity, and microbiota interaction

### 4.1 Pro-inflammatory lipid mediators (eicosanoids)

Lipid mediators are biologically active molecules synthesized from fatty acids that exert diverse physiological effects. PUFAs, such as LA, AA, EPA, and DHA, undergo enzymatic metabolism *via* LOX, COX, or CYP450 monooxygenase enzymes to generate eicosanoids, which are also referred to as fatty acid epoxides (Jing et al., 2025). Eicosanoids encompass PGs, thromboxanes (TXs), LTs, and HETE derivatives. LTs are synthesized *via* LOX pathways, whereas PGs and TXs are produced through COX-mediated oxygenation of AA, collectively exerting predominantly pro-inflammatory effects (Soppert et al., 2020).

Lipid mediators play essential roles in immune regulation, inflammatory responses, vascular tone, and tissue resilience (Wallace, 2019). Eicosanoids exert their biological actions through GPCR expressed across multiple cell populations (Calder, 2020). PGs, lipoxins, and LTs are particularly important for maintaining mucosal integrity; however, under conditions of gastrointestinal epithelial injury, these mediators may also initiate or amplify inflammatory cascades (Wallace, 2019). Due to their rapid catabolism, eicosanoids primarily act in a localized and tissue-specific manner (Calder, 2020).

The gut microbiota directly and indirectly modulates colonic eicosanoid levels. Microbial enzymes facilitate the conversion of fatty acid epoxides to fatty acid diols, altering the concentration and bioavailability of lipid mediators (Huang et al., 2021). Eicosanoids generated *via* CYP450 pathways have been associated with intestinal barrier integrity, colonic inflammation, and colorectal carcinogenesis. Findings from preclinical mouse experiments have shown that changes in colonic CYP eicosanoids mediated by the gut microbiota directly catalyze the hydrolysis of fatty acid epoxides to diols by gut microbes (Jing et al., 2025). A clinical study in patients with ulcerative colitis found that EPA-FFA supplementation reduced mucosal inflammation and regulated gut microbiota composition (Prossomariti et al., 2017).

### 4.2 Specialized pro-resolving mediators

SPMs constitute a broad family of endogenous lipids that play a central role in maintaining inflammatory responses within physiological limits. By restricting excessive neutrophil recruitment, promoting neutrophil apoptosis, and suppressing the local production of pro-inflammatory mediators at the lesion site, they facilitate the controlled resolution of inflammation and help prevent collateral tissue damage (Ortega et al., 2025). Owing to their dual capacity to exert anti-inflammatory effects while actively promoting the physiological resolution of inflammation, SPMs have emerged as notable biological regulators and potential therapeutic targets in inflammatory disorders (Conte et al., 2018; Chen et al., 2025).

SPMs comprise four major families, namely, Rvs, lipoxins, protectins, and maresins (Biełach-Bazyluk et al., 2026). Lipoxins represent the first identified SPM family exhibiting both anti-inflammatory and pro-resolving properties and are biosynthesized in humans from the omega-6 PUFA AA. In contrast, the other SPM families are primarily derived from omega-3 PUFAs, including DHA, EPA, and DPA (Díaz Del Campo et al., 2022).

SPMs are synthesized from LCFA through COX, LOX, and CYP450 enzymatic pathways (Albuquerque-Souza and Dalli, 2025). SPM production relies on coordinated transcellular enzymatic interactions between immune effector cells and resident tissue cells such as epithelial and endothelial cells. This is attributable to the cell type-specific distribution of the enzymes involved in SPM biosynthesis: 15-LOX is predominantly expressed in epithelial cells, COX-2 in endothelial cells, 5-LOX in granulocytes, and 12-LOX in platelets. After exerting their biological effects, SPMs are subsequently inactivated and metabolized by catabolic enzymes such as 15-hydroxy PG dehydrogenase (15-PGDH) (Kang et al., 2025).

SPMs exert their biological effects through GPCRs expressed on the cell surface. However, although these interactions are selective, they do not follow a strict one-to-one pairing model. A single receptor may recognize and transmit signals from multiple SPMs, while an individual SPM can activate more than one GPCR (Zhang Q. et al., 2025). To date, several receptors have been identified in association with SPM signaling, including LTB4R/BLT1, which binds RvE1 and RvE2; CMKLR1 (also known as ChemR23/ERV1), which recognizes RvE1; ALX/FPR2, which interacts with RvD1, RvD3, and LXA4; and GPR32/DRV1, which binds RvD1, RvD3, and RvD5. In addition, GPR18/DRV2 has been described as a receptor for RvD2, GPR37 for PD1, LGR6 for MaR1, and GPR101 for n-3 D-series Rvs (Julliard et al., 2022)

These pro-resolving mediators do not exert their effects by simply suppressing inflammatory pathways. Rather, they actively engage specific resolution programs that are biologically designed to restore tissue homeostasis. Their actions are, therefore, regulatory and restorative, not merely inhibitory (Valente et al., 2022). Within the gastrointestinal tract, SPMs enhance the intestinal barrier function, preserve epithelial integrity, and mitigate mucosal dysfunction (Quiros and Nusrat, 2019). By preventing the persistence of inflammation, these mediators contribute to the restoration and maintenance of tissue homeostasis. The relationship between microbiota and SPMs is bidirectional, as alterations in SPM biosynthesis can reshape microbial composition, while microbial activity may influence the availability and function of SPM(Albuquerque-Souza and Dalli, 2025).

### 4.3 Endocannabinoid and eCB-like lipids

ECS primarily includes type 1 and 2 cannabinoid receptors (CB1 and CB2), lipid-derived endogenous ligands, and the enzymes responsible for the synthesis and degradation of these ligands (Cani et al., 2016). Anandamide (AEA) and 2-arachidonoylglycerol (2-AG) are the primary endogenous endocannabinoids and are synthesized from AA (Pete and Narouze, 2021). AEA is mainly produced from membrane-associated N-arachidonoyl-phosphatidylethanolamine through the action of NAPE-specific phospholipase D, whereas 2-AG is generated from diacylglycerol precursors *via* diacylglycerol lipase isoforms. Both ligands display high affinity for CB1 and CB2 receptors (Pete and Narouze, 2021; Luchicchi and Pistis, 2012).

The expanded ECS, or endocannabinoidome, is important in host–microbiota interactions, digestive function, and energy metabolism (Forte et al., 2020). This signaling lipid system comprises many LCFA mediators and metabolic enzymes that share structural similarities with eCBs and their own receptors. Probiotic use can alter the host’s eCB levels. Furthermore, many commensal bacteria can also synthesize eCB-like metabolites (Cohen et al., 2017).

Endocannabinoid-like lipids, including oleoylethanolamide, palmitoylethanolamide, and linoleoylethanolamide, expand the functional scope of the ECS (Parksepp et al., 2022). Although these molecules bind weakly to classical cannabinoid receptors, they exert significant biological activity through alternative targets such as peroxisome proliferator activated receptor-alpha (PPAR-α), TRPV1, and GPR55 (Rakotoarivelo et al., 2024).

Increasing clinical and preclinical study evidence indicates a strong link between ECS and the gut microbiota (Lamichhane et al., 2021; Tagliamonte et al., 2021; Liu M. et al., 2024). The ECS regulates energy homeostasis, inflammation, intestinal barrier integrity, and immune responses (Hryhorowicz et al., 2021). In particular, oleoylethanolamide and palmitoyl ethanolamide generate anti-inflammatory lipid signals that suppress inflammation and support epithelial integrity (Borrelli et al., 2015). While CB1 activation influences intestinal motility and permeability, CB2 activation primarily attenuates inflammatory cytokine production. Endocannabinoid signaling engages intracellular pathways including nuclear factor-κB (NF-κB), mitogen-activated protein kinases (MAPK), and phosphatidylinositol 3′-kinase-Akt, thereby modulating cytokine synthesis, cell proliferation, and apoptosis (Aloisio Caruso et al., 2025). A randomized double-blind study evaluating the effects of inulin and probiotics on inflammatory parameters indicated that the probiotic–inulin combination reduced the IL-6, LPS, and TLR4 levels and increased the total antioxidant capacity (Liu M. et al., 2024).

Microbial modification of dietary fatty acids alters endocannabinoid bioavailability and affects AEA and 2-AG synthesis. In turn, ECS activity regulates gut barrier permeability, thus influencing microbial translocation and circulating LPS levels (Naughton et al., 2013). A randomized controlled clinical trial in obese individuals observed that the Mediterranean diet affected the endocannabinoid system and increased the *Akkermansia muciniphila* levels independently of weight loss (Tagliamonte et al., 2021).

## 5 Mechanistic crosstalk between the microbiota and lipid mediators

Lipid mediators and gut microbiota engage in a dynamic bidirectional interplay encompassing biochemical and physiological processes (Yu Y. et al., 2019). Microbial communities influence lipid mediator availability through substrate provision, receptor-level signaling modulation, and regulation of immune and barrier functions. Through these interconnected processes, the microbiota supports homeostasis, immune regulation, tissue integrity, and metabolic balance (Xiong, 2025).

### 5.1 Lipid substrate availability and enzymatic conversion

The gut microbiota contributes to fat digestion through two fundamental and independent mechanisms. In the first pathway, the microbiota indirectly enhances the host’s digestive capacity by stimulating pancreatic lipase secretion. In the second pathway, microbially derived lipase enzymes directly participate in the hydrolysis of triglycerides (Yin et al., 2025).

Cholecystokinin (CCK) is a classical enteric peptide hormone known to coordinate gallbladder contraction, pancreatic enzyme release, and gastrointestinal motor activity (Rehfeld, 2025). Beyond its established role in digestive physiology, accumulating evidence indicates that CCK also participates in microbiota-driven metabolic regulation and exerts immunomodulatory effects within the intestinal mucosa (Yin et al., 2025; Saia et al., 2020). In a preclinical study demonstrating that the gut microbiota may regulate the CCK system through microbial metabolites, mice with fructose malabsorption exhibited a significant increase in CCK mRNA expression in the ileum and cecum. This upregulation was observed in parallel with increased populations of *Actinobacteria*, *Bacteroidetes*, and *Lactobacillus johnsonii*. Notably, the finding that propionate can induce CCK gene expression indicates that microbial metabolites may act as mediators in the regulation of the CCK system (Zhang et al., 2019). Moreover, mouse experimental models of endotoxemia have demonstrated that CCK modulates pro-inflammatory cytokine production and increases the colonic expression of tight junction proteins, including occludin, claudin 1, and junctional adhesion molecule A, through the activation of the cholecystokinin 1 receptor (CCK 1R) (Saia et al., 2020).

In a clinical study with healthy individuals, the incretin hormone GLP-1 enhances insulin secretion and suppresses glucagon release in response to nutrient intake. It also delays gastric emptying and promotes satiety (Andersen et al., 2018). However, GLP-1 is not limited to digestive and glycemic control functions and is increasingly recognized as being engaged in bidirectional interactions with the gut microbiota. On the one hand, microbiota-derived metabolites stimulate GLP-1 secretion, and alterations in gut microbial composition can influence its function and circadian rhythm. On the other hand, the regulatory effects of GLP-1 on the gut microbiota appear to involve inflammatory signaling pathways (Zeng et al., 2024). Moreover, a systematic review demonstrated that GLP-1 analogs have been shown to exert significant effects on the composition, richness, and diversity of the gut microbiota (Gofron et al., 2025).

Beyond modulating the host’s pancreatic lipase secretion, the gut microbiota directly contributes to triglyceride hydrolysis through the production of microbial lipases (Yin et al., 2025). Compared with plant- and animal-derived lipases, microbial lipases offer distinct advantages due to their high catalytic efficiency, ease of production, and suitability for genetic engineering. In this context, various strains belonging to the genera *Pseudomonas* and *Bacillus*, along with fungal species such as *Penicillium* and *Trichoderma*, are frequently reported in the literature for their effective lipase production (Abdelaziz et al., 2025). Taken together, these findings highlight the capacity of the gut microbiota to regulate dietary lipid availability and enzymatic processing, ultimately shaping the host’s lipid metabolic efficiency.

### 5.2 Receptor-mediated interactions in the gut microbiota–host axis

The effects of the gut microbiota on the host are largely mediated through receptor-dependent mechanisms. Microbiota-derived metabolites exert their regulatory functions by activating the host’s cell surface and nuclear receptors, thereby shaping the physiological and pathophysiological processes (Chang, 2024). Lipid mediators mediate signal transduction in cells *via* GPCR (e.g., TGR5), nuclear receptors (e.g., FXR and PPAR), and some lipid receptors (Rosenbaum et al., 2009). The microbiota can regulate the activation profile and expression of these receptors by altering the modified lipid ligand pool (Xiong, 2025). Furthermore, receptor-mediated signaling pathways can cause long-term epigenetic and transcriptional changes in immune cells, epithelial cells, or metabolic tissues. This indicates that the interaction between the microbiota and lipid mediators can generate chronic physiological consequences (Lv et al., 2025).

SCFAs exert their biological effects through metabolite-sensing GPCRs expressed on intestinal epithelial and immune cells, including free fatty acid receptor 3 (FFAR3/GPR41), FFAR2 (GPR43), and GPR109A (also known as hydroxycarboxylic acid receptor 2, HCAR2) (Carretta et al., 2021). These receptors are also present in adipose tissue, pancreas, and various immune cell populations. Additionally, acetate and propionate can activate olfactory receptors such as OR51E2 (Olfr78 in mice). This widespread distribution highlights the systemic metabolic and immunomodulatory roles of the SCFA receptors (van der Hee and Wells, 2021). Bile acids derived from both the host and the gut microbiota exert their biological effects primarily through the TGR5 and FXR. Additionally, the vitamin D receptor (VDR), pregnane X receptor (PXR), and constitutive androstane receptor have also been reported to participate in bile acid-mediated signaling (Chang, 2024; Han et al., 2023; Wahlström et al., 2017). Similarly, other bioactive lipid mediators such as lysophosphatidic acid and sphingosine-1-phosphate signal through dedicated GPCR families (LPA1-6 and S1P1-5), which regulate immune cell trafficking, vascular integrity, and inflammatory responses. Additional lipid-derived ligands, including lysophosphatidylinositol and oxysterols, further expand this receptor network, underscoring the central role of GPCR-mediated lipid signaling in host–microbiota communication (Im, 2013).

### 5.3 Immunological and barrier consequences of lipid–microbiota signaling

Interactions between lipid mediators and the gut microbiota critically regulate the integrity of the intestinal epithelium and immune homeostasis. The epithelial barrier maintains homeostasis by selectively controlling permeability between the host and luminal microbes (Quiros and Nusrat, 2019).

The initiation and progression of inflammatory processes are orchestrated by soluble mediators, primarily cytokines, and chemokines. However, inflammation is not limited to protein-derived mediators; lipid mediators that are locally produced at sites of tissue injury and function to restrain excessive leukocyte infiltration and pro-inflammatory signaling also play pivotal roles throughout all phases of the inflammatory response. Notably, LTs and PGs are rapidly generated during the early phase of inflammation, whereas SPMs, such as lipoxins, Rvs, and maresins, are produced at later stages to actively promote the resolution of inflammation and restore tissue homeostasis (Brennan et al., 2021; Schmid et al., 2021).

Lipid mediators also exert potent effects on innate immune cells, including macrophages, DCs, and ILCs. These cells sense lipid signals through TLRs, nuclear receptors, and GPCRs, thereby shaping cytokine production, antigen presentation, and the magnitude of inflammatory responses (Hubler and Kennedy, 2016). Microbiota-derived and modified lipid signals influence the balance between Treg and effector T-cell populations, such as Th17 cells. Favoring regulatory T-cell responses is necessary for sustaining mucosal tolerance and preventing chronic intestinal inflammation (Julliard et al., 2022). Disruption of barrier integrity increases systemic exposure to microbial products, promoting low-grade chronic inflammation, metabolic dysfunction, and immune-mediated diseases (Yang-Jensen et al., 2025).

Lipid mediators, particularly BA derivatives and inflammation-associated lipids, modulate the expression of tight junction proteins such as zonulin, occludin, and claudins. Eicosanoids, which are potent bioactive signaling lipids derived from AA and EPA, are also thought to contribute to the regulation of intestinal barrier integrity (Shi et al., 2023; Morelli et al., 2025). A recent preclinical mice study demonstrated that deficiency of GPR164, a free fatty acid receptor activated by short- and medium-chain fatty acids, increases epithelial proliferation and disrupts Wnt/β-catenin signaling, thereby compromising intestinal barrier integrity. These findings indicate that GPR164-mediated lipid signaling represents a fundamental regulatory mechanism in maintaining barrier integrity and intestinal homeostasis (Ikeda et al., 2025).

## 6 Disease-oriented applications of lipid–microbiota interactions

**FIGURE 3:**
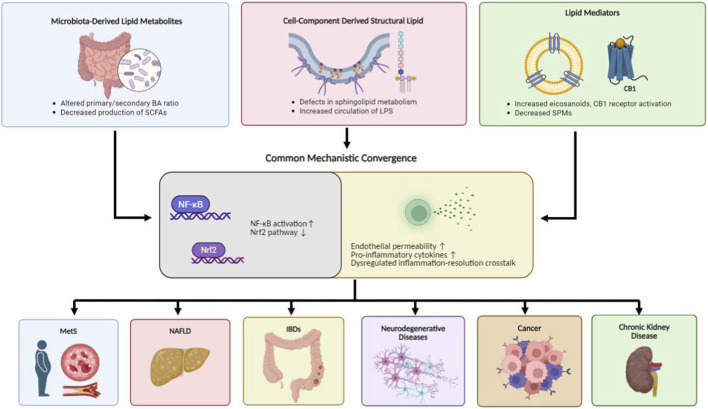
Convergent mechanistic pathways linking lipid dysregulation to chronic disease pathogenesis. BA, bile acid, SCFAs, short-chain fatty acids, Nrf2, nuclear factor erythroid 2-related factor 2, NF-κB, nuclear factor kappa B, SPMs, specialized pro-resolving mediators, CB1, cannabinoid receptor type-1, LPS, lipopolysaccharide, MetS, metabolic syndrome, NAFLD, non-alcoholic fatty liver disease, IBDs, inflammatory bowel diseases.

The role of lipids in interactions between the host and microbiota has become clearer with the development of chemical and lipidomic techniques in recent years. Endogenously synthesized or microbiota-derived biotransformed lipids exert profound influences on the host’s physiology by modulating key metabolic networks and immune signaling pathways. In addition, changes in the cell-component derived structural lipids can stimulate the development of diseases by affecting signaling pathways (Brown et al., 2023). The integrated roles of microbiota-derived, structural, and mediator lipids in chronic inflammation and disease pathogenesis are shown in Figure 3.

### 6.1 Metabolic syndrome

MetS represents a complex modifiable cluster of risk factors that predispose individuals to type 2 diabetes mellitus, cardiovascular diseases, and various adverse health outcomes (Neeland et al., 2024). Numerous metabolic abnormalities of the MetS can be influenced by the quantity and quality of dietary lipids (Basak et al., 2022). Recent evidence indicates that microbiota-derived lipid metabolites, cell-component derived lipids, and lipid mediators also play a significant role in modulating several risk factors related to MetS through their impact on the host’s metabolic pathways (Croci et al., 2021). Moreover, the gut microbiota and its byproducts have been linked to MetS owing to their influence on energy homeostasis, gastrointestinal motility, appetite regulation, carbohydrate and lipid metabolism, and hepatic fat accumulation (Basak et al., 2022).

#### 6.1.1 Microbiota-derived lipid metabolites and MetS

The gut microbiota’s fermentation of fiber produces SCFAs, which can function as signaling molecules by attaching to GPCRs (GPR41 and GPR43). When bound to these receptors, SCFAs regulate the appetite and glucose homeostasis through the release of gut hormones such as GLP-1 and peptide YY (Cani and Van Hul, 2024). SCFAs increase the mitochondrial fatty acid beta-oxidation by activating peroxisome proliferator-activated receptor gamma (PPAR-γ) in colonocytes. Oxidative phosphorylation-induced oxygen consumption helps in the stabilization of hypoxia-inducible factor 1 (HIF1), which increases the expression of genes that are essential for intestinal barrier function, including occludin, zonula occludens, and junctional adhesion molecules. PPARγ activation also exerts anti-inflammatory effects by increasing the expression of anti-inflammatory immunoglobulin A (IgA) and β-defensin and inhibiting NFκB signaling (Overby and Ferguson, 2021). Furthermore, SCFAs function as natural inhibitors of HDAC to epigenetically control gene transcription and chromatin acetylation related to inflammation, apoptosis, fatty acid oxidation, cell differentiation, and cell proliferation (May and den Hartigh, 2023). All these mechanisms are possible pathways of SCFAs on obesity and adipose tissue.

SCFAs have been implicated in the regulation of insulin resistance and type 2 diabetes mellitus, both of which represent key elements of the MetS. It has been reported that propionate can increase insulin release triggered by glucose, protect β-cell mass by reducing trans-differentiation in α-cells, prevent apoptosis, and help proliferation (Mazhar et al., 2023). SCFAs play a crucial role in mitigating chronic low-grade inflammation by modulating cytokine production in immune cells. In particular, butyrate and propionate downregulate the expression of tumor necrosis factor-alpha (TNF-α), IL-6, and nitric oxide, which are key pro-inflammatory mediators released by macrophages as a reaction to LPS (Du et al., 2024). Through these anti-inflammatory effects, SCFAs enhance insulin sensitivity and support metabolic balance. Moreover, butyrate has extra preventive benefits for type 2 diabetes mellitus by inhibiting HDAC. This inhibition activates the nuclear factor erythroid 2-related factor 2 (Nrf2) signaling pathway, thereby augmenting antioxidant defenses and suppressing inflammation, ultimately protecting against diabetes-related oxidative and tissue damage (Tang and Li, 2021). SCFAs also regulate glucose metabolism through the gut–brain neural axis. Propionate increases c-Fos expression and stimulates intestinal gluconeogenesis gene expression through the hypothalamus and neural pathways by activating the GPR41 receptor (Li et al., 2024b). Other effects of SCFAs on glucose metabolism include decreasing gluconeogenesis and hepatic glycolysis in the liver, increasing glucose transporter (GLUT)-4 expression through AMP-activated protein kinase (AMPK) activity, and improving the uptake of glucose in adipose tissue and skeletal muscle (Portincasa et al., 2022).

It is not unexpected that SCFAs are implicated in dyslipidemia and atherosclerosis given the variety of roles they play. It was discovered that butyrate increased the expression of several genes, such as cholesterol 7α-hydroxylase (CYP7A1), low-density lipoprotein receptor (LDLR), and sterol regulatory element binding protein (SREBP2). Increased hepatic expression of SREBP2 and LDLR promoted the hepatic uptake of circulating cholesterol, but increased CYP7A1 expression reduced the blood cholesterol levels by increasing the excretion of BAs in feces (Feng and Xu, 2023). Moreover, it was indicated that acetate induces the phosphorylation of AMPKα, leading to elevated expression of PPAR-α in hepatocytes. This series of events inhibits SREBP-1c expression, thereby downregulating lipogenic gene mRNA and reducing hepatic fatty acid synthesis (Li et al., 2024b). Another effect of SCFAs on atherosclerosis is connected to their regulatory role in inflammation. SFCAs inhibit the production of pro-inflammatory cytokines IL-6 and IL-8 and the formation of foam cells through GPR41/43 activation. They also enhance the production of Treg cells and suppress HDAC, thus suppressing the inflammatory response and, consequently, the development of atherosclerosis (Flaig et al., 2023). Moreover, SCFAs can increase the expression of apolipoprotein A5, which lowers plasma triglyceride levels. While the ability of apolipoprotein A5 to control lipoprotein lipase seems to be its primary mechanism, other modes of action have been suggested, such as decreasing the liver’s release of very low-density lipoprotein (VLDL), improving the uptake of triglyceride containing particles (Zwartjes et al., 2021).

When evaluated in terms of hypertension, SCFAs were thought to be connected to controlling blood pressure. Both hypotensive and hypertensive effects are possible. Blood pressure increases when binding with olfactory receptor-87 (Olfr87) and falls when binding with GPR41. Butyrate may lower the diastolic blood pressure by reducing inflammation (Lange et al., 2023). Moreover, by suppressing the intra-renal renin–angiotensin system mediated by the renal (pro)renin receptor, butyrate may prevent angiotensin II-treated hypertension (Chen et al., 2021).

BAs represent another class of lipid-derived metabolites. TGR5 belongs to the broad family of membrane protein receptors known as GPCRs. Many of the effects on systemic metabolism attributed to BAs are regulated by TGR5, and numerous mechanisms have been proposed for the effects of TGR5 on obesity. Through the cAMP–protein kinase A (PKA)–D2 signaling pathway, TGR5 stimulates the expression of genes linked to thermogenesis and increased energy expenditure. It can enhance mitochondrial function, encourage mitochondrial division, and boost thermogenic gene expression. TGR5 is expressed in the arcuate nucleus, where its activation suppresses the activity of agouti-related peptide (AgRP) and neuropeptide Y (NPY) neurons, thereby contributing to the regulation of appetite through a reduction in food intake (Lun et al., 2024). TGR5 also suppress appetite by promoting gastrointestinal hormone secretion, including PYY and GLP-1. Finally, by inhibiting the activation of the inflammatory pathway in macrophages, the release of inflammatory factors, and the regulation of the ratio of Th17 to Treg cells, TGR5 protects against obesity (Li et al., 2021). FXR, a nuclear receptor expressed on a variety of immune cells, such as DCs, monocytes, macrophages, and NKT cells, also has important effects on the cytokine production and inflammation. In macrophages, FXR activation decreases the release of inflammatory molecules such as IL-1β and IL-6 and suppresses NF-κB activation (Li et al., 2024a).

BAs play a protective function in insulin resistance and type 2 diabetes through nuclear and membrane receptors. BAs activate hepatic FXR, which in turn stimulates the expression of the target gene small heterodimer partner (SHP). By blocking fructose-1,6-bisphosphatase and phosphoenolpyruvate carboxykinase, SHP expression reduces hepatic gluconeogenesis (Gao et al., 2022). Moreover, fibroblast growth factor 19/15 (FGF19/15) is released when secondary BA activates the FXR. FGF19/15 functions as a ligand to enhance glucose tolerance and insulin sensitivity. Additionally, secondary BAs can cure insulin resistance and aberrant glucose metabolism and stimulate muscle energy consumption and intestinal L-cell secretion of GLP-1 (Zhang L. et al., 2021). Enhancing hepatic glycogen synthesis and insulin sensitivity; stimulating insulin secretion; raising energy expenditure in the liver, brown adipose tissue, and skeletal muscle; encouraging thermogenesis and weight loss; and modifying satiety signals in the brain are the main ways that TGR5 regulates glucose and lipid metabolism (Gao et al., 2022).

BAs control metabolic homeostasis and play a role in cardiovascular disorders such as myocardial infarction, diabetic cardiomyopathy, atherosclerosis, arrhythmia, and heart failure. It is believed that BAs that function as signaling mediators and attach to different receptors that influence lipid profile regulation and metabolism. For example, FXR activation is expressed in the cardiovascular system and is linked to preserving appropriate levels of triacylglycerol and cholesterol (Zhang et al., 2023). Furthermore, by activating FXR, they reduce ROS production, trap oxygen free radicals, and reduce myocardial apoptosis, while TGR5 activation enhances Akt signaling, increases nitric oxide availability, and improves endothelial function (Punzo et al., 2024). Recent findings also indicate that BAs could be involved in the regulation of salt-sensitive hypertension. They may reduce blood pressure through several pathways, such as promoting vasodilation and exerting anti-inflammatory actions. In the anti-inflammatory pathway, BAs can directly influence DCs, thereby affecting Treg cell differentiation, inflammasome activity, and the inflammatory cytokine release (O’Donnell et al., 2023). As endogenous vasodilators, BAs control blood pressure and vasomotion by increasing the synthesis of nitric oxide and preventing the release of endothelin-1. Moreover, the trimethylamine-N-oxide (TMAO) pathway is intimately linked to BA metabolism. FXR controls flavin monooxygenase 3 (FMO_3_) activities. Liver FMO_3_ will oxidize trimethylamine (TMA) into TMAO after it enters the liver through the portal circulation. After that, TMAO enters the bloodstream and increases the blood pressure by changing platelet function and lipid metabolism (Yang et al., 2023).

Despite the protective effects of BAs in MetS, the opposite effect may be possible with the change in intestinal microbiota. The ileal absorption of BAs is impaired by changes in the gut microbiota. As a result, there is a decrease in the quantity of colonic primary conjugated BAs and the expression of FXR and FGF19 in intestinal epithelial cells. Dysfunction of the gut microbiota also causes a reduction in the conversion of primary conjugated BAs to secondary BAs in the colon, which impairs TGR5 activation. Thus, the effect of TGR5 activation on the increase in white browning of adipose tissue and GLP-1 was suppressed (Agus et al., 2021).

*Lactic acid bacteria* can also produce intermediates from fatty acids. The most important fatty acids appear to be those derived from oleic or linoleic acids, such as HYA, KetoC, and 13-hydroxy-9-cis-octadecenoic acid (Carneiro et al., 2022). HYA activates GPR40, which induces GLP-1 secretion. Thus, it may participate in appetite suppression and the prevention of obesity (Miyamoto et al., 2019).

#### 6.1.2 Cell-component-derived structural lipids and MetS

Inflammation caused by metabolic endotoxemia is considered one of the key mechanisms contributing to the development of obesity. LPS from the outer membrane of Gram-negative bacteria can move into the systemic circulation when the intestinal barrier is impaired. These LPS molecules interact with TLR-4, triggering downstream inflammatory signaling pathways (Cani and Van Hul, 2024). When LPS binds to TLR-4, the adapter protein myeloid differentiation factor 88 (MyD88) is recruited, initiating a signaling cascade that activates IL-1 receptor-associated kinase (IRAK), tumor necrosis factor receptor-associated factor 6 (TRAF6), and transforming growth factor-activated kinase 1 (TAK1). When TAK1 is activated, NF-κB is released and moves to the nucleus, where it stimulates the transcription of pro-inflammatory cytokines such TNF-α, IL-1, IL-6, and IL-8. LPS can also stimulate the inflammatory response, oxidative stress, cell growth, and apoptosis *via* the MAPK pathway (Wang K. et al., 2024). High levels of LPS in plasma and enhanced inflammation downregulate key genes for lipogenesis and adipose tissue function, such as SREBP1, fatty acid synthase (FASN), fatty acid-binding protein 4 (FABP4), and leptin gene expression. The decreased expression of these factors may impair the adipose tissue’s capacity to handle and store excess lipids (Du et al., 2022). It has been shown that LPS can change the secretion of adipokines such leptin, apelin, and adiponectin in addition to having a direct effect on the adipogenesis processes (Cani and Van Hul, 2024). LPS can also cause insulin resistance and diabetes by impairing insulin sensitivity through the activation of TLRs and stimulation of inflammatory processes (Croci et al., 2021). A systematic review reported that in the majority of the included studies, diabetic participants exhibited higher LPS levels than healthy controls (Gomes et al., 2017).

Inflammatory environment caused by LPS should be regarded as a potential concern for the development or aggravation of atherosclerosis. Through a number of mechanisms, including increasing ROS production, upregulating adhesion molecule expression, and encouraging the movement of inflammatory cells to the affected site, LPS may stimulate or accelerate the development of atherosclerosis (Gorabi et al., 2022). LPS is a pro-atherogenic molecule that enhances LDL oxidation *via* its pro-oxidant activity, primarily through the activation of nicotinamide adenine dinucleotide phosphate oxidase 2 (Nox2), a key cellular source of ROS (Violi et al., 2023a). LPS increases the infiltration of inflammatory cells in cholesterol plaques by inducing the production of inflammatory mediators. These inflammatory cells, which contribute to the development of atherosclerosis, comprise neutrophils, monocytes, selectins, and integrins (Al Samarraie et al., 2023). Additionally, LPS promotes the development of thrombus at the site of atherosclerotic plaque rupture or erosion by binding to TLR-4 in a variety of cell types, including endothelial cells, monocytes, neutrophils, and platelets. Von Willebrand factor (vWf) and factor VIII (FVIII) are released by TLR-4 activation in endothelial cells *via* increasing the secretion of tissue factor (TF) and producing and secreting Weibel-Palade bodies (WPb). In monocytes, TLR-4 causes TF release to be upregulated. TLR4 signaling in neutrophils initiates both cathepsin G-mediated platelet activation and neutrophil extracellular traps (NETs). Platelet activation is caused by LPS-mediated TLR-4 signaling (Violi et al., 2023a). Endothelial activation and hypertension are also linked to TLR4 activation. Exposure to LPS increases oxidative stress, leading to reduced nitric oxide bioavailability and endothelial dysfunction, while the activation of inflammatory pathways contributes to vascular inflammation (Grylls et al., 2021).

Bacterial sphingolipids and their metabolites can lead to systemic host effects. Ceramide and sphingosine-1-phosphate (S1P), two sphingolipid metabolites, are potent signaling chemicals that regulate a number of host cell processes, including as inflammation, differentiation, apoptosis, and proliferation (Heaver et al., 2018). Bioactive sphingolipid metabolites such as ceramide and S1P have recently been recognized as lipids that accumulate in obesity and act as key molecular regulators in metabolic diseases (Green et al., 2021).

#### 6.1.3 Lipid mediators and MetS

Oxylipins refer to lipid mediators formed through the PUFAs oxidation by enzymatic or non-enzymatic mechanisms. Chronic inflammation in obesity is associated with changes in circulating eicosanoids and oxylipins such as PGE_2_, PGF_2_α, 8-iso-PG A_1_, 12- hydroxyheptadecatrienoic acid, 9,10-dihydroxyoctadecenoic acid, and 12,13-dihydroxy octadecenoic acid several of which have shown predictive value for insulin resistance, type 2 diabetes, and obesity-related complications, while others, such as SPMs including RvD_2_ and maresin-1, are reduced and associated with impaired resolution of inflammation (Misheva et al., 2022).

SPMs can inhibit the production of pro-inflammatory cytokines, block neutrophil activation and recruitment, and induce non-phlogistic (anti-inflammatory) activation of macrophage engulfment and removal of apoptotic inflammatory cells and debris. Important information from preclinical and clinical studies has highlighted the roles and impact of SPMs on oxidative stress and inflammation-related MetS (Dubé et al., 2022). However, reduced SPMs’ levels are closely associated with obesity-related inflammation, which prolongs unresolved inflammation and may exacerbate metabolic diseases (Kim and Shin, 2024). Interestingly, a randomized controlled study demonstrated that even a slight reduction in body weight among individuals with MetS enhanced the capacity of their neutrophils to produce RvE1 in response to stimulation (Barden et al., 2019). Moreover, persistent and unchecked inflammation is now widely acknowledged as one of the primary underlying processes linking obesity to systemic insulin resistance. SPMs can regulate insulin signaling pathways and improve insulin sensitivity and glucose absorption by activating IRS-1/PI3K/Akt and upregulating the expression of PPAR-γ and GLUT-4 (Liu et al., 2024c). These mediators also have a protective role in atherosclerosis. SPMs reduce rolling and adhesion of neutrophils and monocytes, promotes plaque stability, macrophage prototype M2 activation, collagen synthesis, and efferocytosis, which shrinks the necrotic nucleus (Salazar et al., 2022).

Endogenously produced cannabinoids also influence development of MetS. ECS plays an important part in several metabolic regulation mechanisms. Appetite, food intake, eating motivation, and energy balance are all impacted by the central nervous system’ ECS. By influencing cell signaling pathways and the synthesis and breakdown of hormones and enzymes, the ECS can affect feeding regulation in both the central nervous system and the periphery. Therefore the overactivation ECS and heightened activation of CB1 contribute to excessive energy intake, enhanced lipogenesis, and an elevated risk of obesity and MetS (Dörnyei et al., 2023). Furthermore, increased levels of endocannabinoids, specifically 2-AG, in adipose tissue have been linked to the insulin resistance development. It has been shown that local increases in tissue 2-AG levels and the subsequent hyperactivity of the CB1 receptor are closely associated with decreased skeletal muscle glucose metabolism, increased abdominal obesity, and increased lipid transport to the liver, which results in an accumulation of free fatty acids in hepatocytes, which is linked to the development of insulin resistance. Thus, it may be said that the ECS controls the metabolism of carbohydrate and lipids (Simankowicz and Stępniewska, 2025). However, compared to CB1, CB2 receptor activation exerts anti-inflammatory effects, may enhance insulin sensitivity, and offers potential metabolic protection across immune cells, adipose tissue, liver, and pancreatic β-cells (Serefko et al., 2025).

### 6.2 Non-alcoholic fatty liver disease

Non-alcoholic fatty liver disease (NAFLD) is a significant liver condition affecting roughly 25% of adults worldwide, contributing to considerable health, social, and economic consequences. It is recognized as a multisystem disorder and represents the MetS manifestation related to the liver (Lazarus et al., 2022). The complex pathophysiology of NAFLD is still mostly unknown. However, according to the multiple hit theory, oxidative stress, inflammation, lipotoxicity, and mitochondrial dysfunction in hepatic tissue are caused by the adipokines, gut microbiome, and insulin resistance (Ji et al., 2019).

#### 6.2.1 Microbiota-derived lipid metabolites and NAFLD

Through a number of mechanisms, SCFAs are essential for controlling the course of NAFLD. For instance, SCFAs can lower cholesterol through inhibiting SREBP-1–mediated cholesterol synthesis, enhancing CYP7A1 expression to promote its conversion to BAs, and regulating transporters such as ATP-binding cassette transport proteins G5 and G8 (ABCG5/8) to increase cholesterol efflux. They also activate GPR43 to strengthen the leptin response, thereby further suppressing hepatic lipogenesis and cholesterol production (Li et al., 2024c). Hepatic fat accumulation is impacted by SCFAs. For example, acetate generated by gut microbiota reduced enterocytes’ production of chylomicrons and increased lipid oxidation by activating the AMPK-peroxisome proliferator-activated receptor gamma coactivator-1 alpha (PGC-1α) and PPAR-α pathway. Hepatic inflammatory responses are decreased by SCFAs. SCFAs help maintain the integrity of intestinal integrity by reducing the levels of circulating LPS and preventing the migration of harmful substances like ethanol. SCFAs also help attenuate inflammation by suppressing key inflammatory pathways. Through GPR41/43-mediated signaling, they further inhibit pro-inflammatory cytokine production, supporting their potential as therapeutic agents for controlling hepatic immune dysregulation and inflammation in NAFLD (Zhang S. et al., 2021). Moreover, SCFAs reduce the progression of NAFLD by inducing liver autophagy through the PPARγ-UCP2-AMPK pathway, which could be a novel treatment approach (Li et al., 2024c).

As signaling molecules, BAs are most likely to improve NAFLD *via* FXR and TGR5. Activation of FXR reduces BA synthesis, enhances BA efflux, and suppresses lipogenic and gluconeogenic genes, thereby lowering hepatic triglyceride accumulation and improving insulin sensitivity. In addition, FXR and TGR5 signaling modulate inflammatory pathways by inhibiting the nucleotide-binding and oligomerization domain-like receptor family pyrin domain-containing 3 (NLRP3) inflammasome assembly in macrophages (Li Z. et al., 2023). Activation of TGR5 may also indirectly protect against NAFLD through increased energy expenditure and anti-obesity effects. Type 2 iodothyronine deiodinase is activated when TGR5 activation increases cAMP levels. This activation facilitates the transformation of thyroxine (T4) into triiodothyronine (T3), the more active form. T3 then increases the production of uncoupling protein 1 (UCP-1), which increases energy expenditure even further. However, despite the protective function of BAs through the aforementioned pathways, dysregulated BA signaling is thought to take part in the development and progression of NAFLD (Wei et al., 2024). There is a reduction in microorganisms that change the main BAs into secondary BAs in NAFLD. This leads to additional disruption of the gut microbiota and a decrease in the activation of BA receptors by secondary BAs (Park et al., 2022).

#### 6.2.2 Cell-component-derived structural lipids and NAFLD

Cell-component-derived LPS is another factor engaged in the pathophysiology of NAFLD. Normally, LPS reaches the liver *via* the portal vein and undergoes detoxification there. However, when small intestinal bacterial overgrowth (SIBO) occurs or intestinal permeability increases, the amount of LPS reaching the liver increases (An et al., 2022). LPS contributes to hepatic injury by binding to LPS-binding protein and CD14, which subsequently activates TLR-4, triggering inflammation and elevating intestinal permeability. These processes jointly play a crucial part in the beginning and development of NAFLD (Gruzdev et al., 2024). All liver cell types, including Kupffer cells, hepatocytes, hepatic stellate cells, and cholangiocytes, express TLR-4. The development of fibrosis and the activation of fibrogenic cells are linked to its expression on these cells. Hepatic stellate cells can be activated by LPS, and Kupffer cells significantly amplify this process by generating transforming growth factor beta (TGF-β) and making hepatic stellate cells more sensitive to TGF-β (An et al., 2022). Furthermore, When LPS binds to TLR-4, it activates downstream signaling pathways that stimulate platelet granule secretion. This process enhances platelet activation and aggregation by increasing the production of eicosanoids such as TXA_2_ and F2-isoprostanes, along with reactive oxidant species generated by Nox2 (Violi et al., 2023b).

A deeper comprehension of the function of sphingolipids in fatty liver disease has arisen as a consequence of the enormous progress made in the field of lipidomics over the past 20 years. Hepatic steatosis is caused by excessive buildup of ceramides in the liver. It has been shown that aberrant ceramide accumulation in NAFLD has growing pathogenic implications, such as encouraging apoptosis, causing insulin resistance, increasing endoplasmic reticulum stress and mitochondrial oxidative stress, and, eventually, leading to steatosis, inflammation, and fibrosis (Zhu et al., 2023). Ceramide concentration and elevated hepatic inflammation have complex and reciprocal interaction. Ceramides can thereby worsen hepatic inflammation by triggering TLR and inflammasome signaling pathways, whereas *de novo* production of ceramides may be increased through TLR-dependent mechanisms (Ramos-Molina et al., 2024). Lysophospholipids have also been identified as possible causes of NAFLD, including lysophosphatidylcholine, lysophosphatidic acid, lysophosphatidylinositol, and lysophosphatidylethanolamine (Papadopoulos et al., 2022a). In an *in vivo* animal model, the results of a study showed that Western diet-induced NAFLD leads to early and progressive alterations in the mitochondrial lipid profile that are particularly characterized by decreased cardiolipin and increased phosphatidic acid. These mitochondrial lipid changes occur alongside increasing oxidative stress, indicating that they may play an early contributing role in NAFLD progression (Durand et al., 2021).

#### 6.2.3 Lipid mediators and NAFLD

Different kinds of oxylipins, such as pro-inflammatory PGs and anti-inflammatory Rvs, have been demonstrated to have conflicting effects in NAFLD (Misheva et al., 2022). For example, LOX metabolites of AA (HETE, 8-HETE, 11-HETE, and 15-HETE) and LA (9-hydroxyoctadecadienoic acid (HODE), 13-HODE, 9-oxo-octadecadienoic acid (oxoODE), and 13-oxoODE) are markedly elevated in non-alcoholic steatohepatitis patients. Additionally, they have higher amounts of AA cytochrome P45 metabolites, such as 11–12 dihydroxyeicosatrienoic acid (DHET) and 14–15 DHET, which are further increased in fibrosis (Béland-Bonenfant et al., 2023). However, in a cross-sectional study evaluating the CYP derivative oxylipins for their potential hepatoprotective effects, the total epoxyeicosatrienoic acids (EET) and total DHET acids were discovered to be substantially lower in NAFLD than in controls (Arvind et al., 2020). Given the high prevalence of NAFLD and the lack of well-validated diagnostic biomarkers, there is a clear need for reliable tools to monitor early metabolic changes in the disease. Although existing studies are promising, most involve small cohorts and lack validation, highlighting the need for large, multi-center trials to assess the accuracy of oxidized fatty acid-based biomarkers. Additionally, the limited stability of oxylipins in biological samples poses a challenge, as sample collection and handling can significantly influence their measured levels and restrict their diagnostic utility (Masoodi et al., 2021).

EPA, docosapentaenoic acid (DPA), and DHA, along with their bioactive derivatives, exert protective effects across numerous diseases, including NAFLD. Evidence indicates that the n-3 fatty acid-derived SPMs (Rvs, protectins, and maresins) share common mechanisms, such as suppressing inflammation, lowering hepatic lipogenesis, and enhancing insulin sensitivity (Maciejewska-Markiewicz et al., 2021).

Following receptor engagement, D-series Rvs (RvD1) uses a variety of signaling pathways to contribute to the control of liver disease. These processes include promoting the release of anti-inflammatory cytokines, reducing leukocyte infiltration, inducing leukocyte apoptosis, enhancing efferocytosis and M2 polarization of macrophages, and inhibiting the release of pro-inflammatory cytokines. To lessen oxidative stress reactions, RvD1 can increase the expression of antioxidants such NADPH quinone dehydrogenase 1, superoxide dismutase, and glutathione and decrease the expression of the oxidant malondialdehyde. Additionally, RvD1 activates the thioredoxin-2 (TRX2) signaling pathway, which restores mitochondrial function and encourages mitochondrial autophagy (Yang et al., 2022). E-series Rvs can improve glucose metabolism and increase insulin sensitivity. Protectin has been shown to restrict the NF-κB pathway through AMPK activation, thereby preventing lipid accumulation and reducing LPS-induced adipocyte inflammation. It has also been shown that maresins activate AMPK and its downstream components linked to autophagy and fatty acid oxidation. In hepatocytes, this activation increases AMPK phosphorylation, which eventually lowers lipid accumulation and endoplasmic reticulum stress (Kim and Shin, 2024). However, obese individuals show an imbalance in pro-resolving the lipid mediators’ production, indicating that restoring these mediators’ levels could help counteract steatohepatitis-related inflammation (Clària et al., 2021).

Endocannabinoid signaling is intimately associated with NAFLD and controls energy homeostasis. Dysregulation of the ECS, namely, overactivation of CB1 receptors, influences the development of non-alcoholic steatohepatitis, hepatic steatosis, and insulin resistance. In the liver, CB1 activation promotes *de novo* lipogenesis and fat storage (Simankowicz and Stępniewska, 2025). Activation of hepatic CB1 receptors stimulated liver lipogenesis and promoted the accumulation of MUFAs. CB1 activation in hepatic stellate cells increases the so-called-endocannabinoids secretion, leading to fibrosis and collagen accumulation. CB1 activation in Kupffer cells enhances inflammation by activating the NF-κB and NLRP3 pathways and increasing pro-inflammatory cytokine production. This activation also triggers hepatocyte apoptosis (Mboumba Bouassa et al., 2022).

### 6.3 Inflammatory Bowel diseases (IBDs)

Crohn’s disease and ulcerative colitis, which phenotypically cover a range of inflammatory bowel disorders (IBDs), have spread around the world during the past several decades because of the more westernized lifestyle and dietary practices (Adolph et al., 2022). Despite the fact that the precise cause of IBDs is still uncertain, it appears to be maintained in genetically vulnerable patients by a compromised immune system against gut microbes. Both innate and adaptive immune responses are dysregulated in this aberrant immune response (Saez et al., 2023).

#### 6.3.1 Microbiota-derived lipid metabolites and IBDs

SCFAs and IBDs have complicated interactions that involve immune responses, gut microbiota, and the integrity of the gut epithelial barrier. By encouraging the creation of mucus and strengthening the tight connections between epithelial cells, SCFAs help to maintain the intestinal barrier. SCFAs are also involved in intestinal tissue healing mechanisms. By promoting the proliferation and differentiation of epithelial cells, they help mend damaged tissues caused by inflammation in IBDs (Shin et al., 2023). By controlling the innate immune sensors TLRs and NLRP3 inflammasomes, SCFAs slow the development of inflammatory bowel disease. Additionally, development of IBDs is significantly influenced by several cytokines, including IL-10 and IL-17. By modifying innate immunity’s recognition function, SCFAs can stop the emergence of excessive immunological responses. They can also preserve the barrier of the intestines by stimulating the synthesis of IL-10 and suppressing that of IL-17 (Zhang Z. et al., 2022). A systematic review analyzing clinical evidence on the therapeutic and immunological effects of SCFAs in IBDs reported that especially butyrate is a key regulator of intestinal inflammation and could be used as the target for therapies aimed at boosting SCFAs-producing bacteria (Ventura et al., 2024).

Patients with IBDs have aberrant microbial composition and reduced microbial diversity, as evidenced by the enrichment of phylum *Proteobacteria* and the depletion of phylum *Firmicutes*, which includes bacteria participating in BA metabolism. BAs transformation is impeded by dysbiosis. Therefore, in IBDs, the concentration of primary and conjugated BAs is increased at the expense of secondary BAs (Yang et al., 2021). There is increasing evidence that the altered primary and secondary BA composition seen in IBD patients may cause pro-inflammatory mucosal responses by acting differently on immunological and epithelial cells than they do in healthy individuals. Multiple BA-responsive receptors that are highly expressed in intestinal tissues are engaged by primary and secondary BAs, which contain derivatives of oxo and iso BA. These receptors are present not only on gut epithelial cells but also on various immune cell types (monocytes/macrophages, Th17 cells, natural killer cells, and DCs *etc.*), indicating that they modulate inflammatory reactions *via* cell type-specific effects on the mucosa of the gut and may influence key pathways involved in IBD, including apoptosis, autophagy, and inflammasome activation (Thomas et al., 2022). However, by altering the activity of retinoid-related orphan receptor (RORγt), novel families of BAs, including several oxo-derivatives, are becoming new factors that control intestinal immunity. These oxo-derivatives of BAs can function as inverse agonists for RORγt, thus directly reducing inflammation, especially on Th17 cells (Biagioli et al., 2021).

#### 6.3.2 Cell-component-derived structural lipids and IBDs

The inflammatory molecule LPS is extensively researched in IBD. The gut is the first location where LPS can cause inflammation; both gut microbiota and gut-associated lymphoid tissue (GALT) are impacted by LPS and become more inflammatory. The alterations in gut microbiota and GALT caused by LPS are comparable to those observed in IBD: microbiota becomes less diverse, and GALT Treg become fewer in number, while Th17 and Th1 lymphocytes proliferate. TLR-4 activation also activates the innate immune system, and direct injury to the epithelium causes further inflammation (Candelli et al., 2021). In a translational study analyzing human tissue biopsies, a total of 37 resected specimens were analyzed, including 20 normal colon tissues and 17 colitis tissues. The results showed that phosphoinositide-3-kinase regulatory subunit 3 (PIK3R3) was significantly upregulated while zonula occludens-1 (ZO-1) decreased during LPS-induced inflammation. These findings indicate that ZO-1 expression is regulated by PIK3R3 *via* the NF-κB pathway, contributing to impaired intestinal barrier integrity in inflammatory bowel disease (Ibrahim et al., 2020).

In IBD, inflammation has also been linked to disrupted sphingolipid metabolism. Early IBD episodes cause ceramide synthases to be overexpressed, which increases the production of ceramide species. Cell-cycle arrest, differentiation, and apoptosis can all be triggered by increases in ceramide. Thereafter, different ceramide pools can be phosphorylated into S1P and deacylated to produce sphingosine. G-protein-coupled S1P receptors, which are also significantly expressed during inflammation, mediate S1P signaling through autocrine to paracrine communication. This increases S1P signaling to support pro-inflammatory cytokine release, cell survival, and proliferation (Espinoza and Snider, 2024). Moreover, increases in the production of sphingolipids, especially ceramide, are recognized to lead to endoplasmic reticulum stress in a variety of tissues. Endoplasmic reticulum stress is recognized for playing a part in IBD, and it is closely related to the innate immune response (Doll and Snider, 2024).

Patients with IBD had higher amounts of lyso phosphatidylethanolamine (LysPC) and lyso phosphatidylserine (LysoPS) among lysoglycerophospholipids in their blood and feces. The expression of ZO-1 and occludin in intestinal epithelial cells is reduced by bacteria-derived LysPC, indicating that dysbiosis-mediated LysoPC compromises the integrity of epithelia. Through GPR34-mediated stimulation of signal transducer and activator of transcription 3 (STAT3) signaling, LysoPS produced by apoptotic neutrophils stimulates the production of IL-22 in 3 ILCs and promotes epithelial tissue healing (Kayama and Takeda, 2023). Alternatively, CL may contribute to IBD by regulating mitochondria-dependent apoptosis. When CL becomes oxidized under increased ROS and oxidative stress, cytochrome c is released from the mitochondria into the cytoplasm, activating caspase signaling and triggering apoptosis, a process which is shown to be elevated in IBD (Boldyreva et al., 2021).

#### 6.3.3 Lipid mediators and IBDs

The importance of lipid mediators in the etiology and management of IBD is becoming more widely acknowledged. PGs are important modulators of inflammatory cell infiltration and vascular responses. PGE2 has pro-inflammatory effects, potentially *via* the control of the IL-23/IL-17 axis in IBD. Under some conditions, both pro-inflammatory and anti-inflammatory actions are possible with PGD2. For example, elevated lipocalin-type PGD synthase may make inflammation worse in ulcerative colitis. On the other hand, PGD2 also protects against IBD. Through the release of PGD2, nicotinic acid controls the expression of repressed pro-inflammatory genes in macrophages. By controlling the generation of cytokines, PGE2 affects the function of the intestinal epithelial barrier. PGE2 inhibits the release of TNF-α in macrophages, interferon-gamma (IFN-γ) in NKT, and IL-12 from developing DCs. Conversely, PGE2 can encourage group-3 ILCs to generate IL-22. The intestinal barrier may be disrupted by these pro-inflammatory cytokines (Huang et al., 2021). However, AA produces the anti-inflammatory lipid mediators lipoxin A4 and lipoxin B4, which reduce inflammation in IBD (Yan et al., 2023). In a clinical study evaluating the expression of oxylipins in IBD, TXB2, PGE2, LTB4, C4, D4, and E4 levels, 15-epi-lipoxin A4 and lipoxin B4 were shown to be significantly higher, and 18-hydroxy-EPA and 6-keto-PGF1α levels were found to be significantly lower in the Crohn’s and ulcerative colitis mucosa in the colon than in the control group (Ben-Mustapha et al., 2024). In another clinical study, increased plasma 6-epi-lipoxin A4 and 2-arachidonyl glycerol and decreased DHA in Crohn’s disease were noted as findings that distinguished patients from controls (Ben-Mustapha et al., 2023). Furthermore, when evaluating human longitudinal disease activity, TXB2, LTB4, and 9S-HODE levels tended to be higher in the active phase of Crohn’s disease, whereas 12S-hydroxyeicosa tetraenoic acid levels were significantly higher in the inactive phase, according to a comparison of the active and inactive disease phases. In ulcerative colitis, PGE2 and 16RS-HETEs levels were significantly elevated in the active phase, while lipoxin A4 5S and 6R levels were significantly higher in the inactive phase (Kikut et al., 2022).

SPMs were able to decrease leukocyte recruitment and regulate the release of pro-inflammatory cytokines in the context of IBD. This indicates that these substances play a significant part in the formation of the inflammatory response, the improvement of the clinical characteristics, and the reduction of tissue damage (Pascoal et al., 2022). By inhibiting the production of the pro-inflammatory cytokine TNF-α, RvE1 prevents the beginning of colitis and lessens the tissue damage and gene expression caused by leukocytes. By preserving intestinal homeostasis, reducing inflammation, and fortifying tight junctions, RvD1 provides protection against IBD. Additionally, RvD1 inhibits the intestinal epithelium’s mesenchymal transition and stops IBD-related fibrosis. Maresin 1 suppresses the NF-κB pathway and lowers the levels of IL-1β, TNF-α, IL-6, and IFN-γ. Additionally, maresin 1 maintains the mucosal barrier, enhances tight junction protein production, and controls colonic inflammation *via* regulating the Nrf2 and TLR4/NF-kB pathways (Yan et al., 2023).

Numerous physiological systems, such as intestinal homeostasis, gastrointestinal motility, and immunomodulation of inflammation in IBD, are also significantly influenced by the ECS (Hryhorowicz et al., 2021). The components of the ECS and the broader endocannabinoidome play roles in numerous gastrointestinal processes, including the control of food intake and satiety, modulation of nausea and vomiting, regulation of gastric secretion and gastroprotection, coordination of gut motility and visceral sensitivity, attenuation of intestinal inflammation, maintenance of epithelial barrier integrity, and regulation of immune tolerance within the gastrointestinal tract. Therefore, managing these mechanisms may be crucial for preventing IBD (Hryhorowicz et al., 2021). However, there is substantial inconsistency across studies regarding the expression and distribution of ECS components in IBD. Although CB1 and CB2 are present in various epithelial, immune, and neural cell types, their regulation during inflammation and the changes in the endocannabinoid levels such as 2-AG and AEA remains conflicting (Ambrose and Simmons, 2018). Providing direct translational evidence from human biopsy specimens, a study comparing the CB1 and CB2 receptors’ relative expression in human biopsy specimens from IBD with unrelated controls showed a significant decrease in CB1 expression only in patients diagnosed with ulcerative colitis compared to that in controls (Wolyniak et al., 2024). In a clinical study evaluating the changes in endocannabinoids, AEA, and 2-AG in adolescents with Crohn’s disease over the course of the disease, it was shown that the 2-AG levels remained stable across the acute phase, treatment, and remission and did not differ from that in controls, whereas AEA concentrations stayed consistently lower than that in the control group’s at all time-points (Bochenek et al., 2024). In another clinical study, patients with Crohn’s disease and ulcerative colitis had higher levels of AEA and oleoylethanolamide in their plasma, while patients with Crohn’s disease had enhanced levels of 2-AG (Grill et al., 2019). Cherkasova and colleagues interpreted the different results found in the studies as increased CB receptor expression and greater endocannabinoid production in mild types of colitis but decreased CB1/CB2 expression and decreased endocannabinoid levels in mild forms of IBD-related colon inflammation (Cherkasova et al., 2021). Both CB1 and CB2 receptors that participate in immune responses are expressed by plasma cells and macrophages in the large intestine. Activated macrophages, monocytes, and DCs produced more endocannabinoids in the inflammatory gut, which may lessen the increased permeability. However, by activating CB1 receptors, increased endocannabinoid levels can cause an increase in inflammation-related intestinal permeability and hypoxia that is concentration-dependent (Cherkasova et al., 2021).

### 6.4 Neurodegenerative diseases

Neurodegenerative disorders are a diverse range of complex diseases marked by the gradual degeneration of various nervous system regions and loss of neurons. With an increasing rate of incidence, neurodegenerative diseases constitute a major global health concern. A complex interplay among genetic, epigenetic, and environmental factors has been proposed, even though the precise pathophysiology of the neurodegenerative diseases remains unknown (Agnello and Ciaccio, 2022).

#### 6.4.1 Microbiota-derived lipid metabolites and neurodegenerative diseases

A wide range of microbially derived molecules released within the digestive tract can influence the gut–brain communication and reach multiple organ systems, including the nervous system. For example, SCFAs inhibit the production of inflammatory cytokines, stimulate T-cell proliferation in various ways, restore microglial activity and maturation, and induce phenotypic changes in microglia to reduce inflammation (Singh M. P. et al., 2022). By modulating the synthesis and function of neurotransmitters and their receptors, SCFAs may affect neurotransmission in the brain. Many neurotransmitters, such as glutamate and gamma-aminobutyric acid (GABA), have been shown to be modulated by butyrate. By regulating the glutamate receptor expression and neurotransmitter release, butyrate may affect the glutamatergic system, which, in turn, may alter synaptic signaling and neuronal excitability. Butyrate may also affect GABA-mediated inhibitory neurotransmission, which is essential for maintaining the brain’s balance between excitation and inhibition (Missiego-Beltrán and Beltrán-Velasco, 2024). Other proposed mechanisms for the protective role of SCFAs against neurodegenerative diseases are as follows: restoring age-related alterations in gut microbiota, maintaining intestinal and brain barrier integrity, regulating the release of neurotransmitters (serotonin, dopamine, and GABA production), and neurogenesis through the release of hormones such as GLP-1, peptide YY and leptin (Guo et al., 2025). Moreover, SCFAs decrease cortisol release by preventing the hypothalamic–pituitary–adrenal (HPA) axis from being overly activated. SCFAs lessen the stress reactions and the strain on the neurological system by reducing the cortisol levels (Choe, 2025).

The immune, neurological, and endocrine routes are the main ways that gut-derived BAs interact with the brain. BAs can release GLP-1 by directly activating FXR and TGR5 on enterocytes. GLP-1 can penetrate the blood–brain barrier and bind to certain receptors to exert anti-inflammatory and neuroprotective effects on the central nervous system. In the immune pathway, by controlling the Th17/Treg ratio, BAs metabolites can preserve immunological equilibrium. Additionally, BAs can increase the IL-10/IL-12 ratio and prevent neuroinflammation by activating TGR5 to decrease NF-κB activity and encourage M2 macrophage polarization (Wang S. et al., 2023). In line with these mechanisms, BAs also exhibit neuroprotective actions across major neurodegenerative disorders. In Alzheimer’s disease, they reduce hippocampal beta amyloid protein (Aβ) production and protect neurons from mitochondrial toxicity. In Parkinson’s disease, BAs limit ROS formation and lipid peroxidation, thereby decreasing neuroinflammation. In Huntington’s disease, they help alleviate endoplasmic reticulum stress. In amyotrophic lateral sclerosis, BAs slow the functional decline by reducing oxidative stress and neuroinflammatory signaling (Grant and DeMorrow, 2020). Therefore, BAs are increasingly being recognized as a potential therapeutic approach for protecting neurons. Hydrophilic BAs, especially tauroursodeoxycholic acid (TUDCA), have exhibited significant anti-apoptotic and neuroprotective properties in recent years. Several experimental and clinical findings indicate their potential therapeutic application as disease-modifiers in neurodegenerative illnesses (Khalaf et al., 2022).

#### 6.4.2 Cell-component-derived structural lipids and neurodegenerative diseases

Two important pathophysiological features of neurodegenerative diseases are oxidative/nitrative stress and inflammation, which can be caused by increased intestinal permeability and pathobiont abundance. The blood–brain barrier is harmed by these alterations because they cause an excessive flow of LPS and other products of bacteria into the blood, which in turn causes persistent systemic inflammation (Kalyan et al., 2022). When LPS binds to and stimulates the TLR-4 receptors on glial cells, it causes neurodegeneration *via* increasing neuroinflammation. Two signaling routes, namely, the MyD88-dependent route and the MyD88-independent pathway, are phosphorylated when LPS stimulates TLR-4. Following this activation, several pro-inflammatory cytokines that are required to initiate innate immune responses are released, which in turn encourages neuroinflammation (Singh S. et al., 2022). Memory impairment is caused by neuronal injury and synaptic loss brought on by chronic neuroinflammation. As a result, these changes have a detrimental impact on mitochondrial function (Kalyan et al., 2022).

Defects in sphingolipid metabolism may be participating in the underlying mechanisms and progression of neurodegenerative disorders. Data from human clinical observation indicate that plasma S1P levels were shown to be significantly lower and monohexylceramide and lactosylceramide levels were shown to be significantly higher in the neurodegenerative patient group than in the control (Oizumi et al., 2022). Ceramide has an apoptotic effect on neuronal cells and changes the molecular phenotype of astrocytes and microglia to one that is pro-inflammatory and promotes the production of inflammatory proteins. S1P, on the other hand, tends to cause morphological alterations in glial cells and contributes to the maintenance of neuroinflammation while protecting neuronal cells (Ayub et al., 2021). Alterations in sphingolipid metabolism following the onset of neurodegenerative disorders may also contribute to disease progression. In Alzheimer’s disease, Aβ accumulation may increase the expression of ceramide synthase 2, an enzyme involved in the ceramide *de novo* synthesis. The disrupted balance between ceramides and S1P may promote neurodegeneration in the cerebral cortex. Furthermore, oligodendrocyte destruction and demyelination have been connected to increased ceramide synthesis and accumulation in active multiple sclerosis (MS) lesions (Alaamery et al., 2021).

As a bioactive phospholipid and an essential regulator of adult neural system development, lysophosphatidic acid has been proposed as a key contributor to neurodegenerative disease processes (Dedoni et al., 2024). The progression of these disorders is strongly driven by fibrillary deposits of the highly conserved and thermostable protein α-synuclein (α-Syn). Owing to their ability to directly bind α-Syn and inhibit its aggregation, lysophospholipids may represent promising therapeutic candidates for preventing neurodegeneration and limiting the cell-to-cell propagation of toxic aggregates (Karaki et al., 2022).

#### 6.4.3 Lipid mediators and neurodegenerative diseases

AA-derived eicosanoids, such as PGs, LTs, and HETEs, have been identified and linked to neurodegenerative diseases (Chiurchiù et al., 2022). In Alzheimer’s, Aβ production is stimulated by TXA2, isoprostanes, 5-HETE, LTB4, LTC4, and LTD4. However, PGF2α′s role in Alzheimer’s disease appears to be indirect and only marginally Alzheimer’s-promoting, especially in the very early stages of the disease (Biringer, 2019). In a Parkinson’s disease-related study, patients exhibited elevated levels of two eicosanoids—AA and 13-hydroxy-octadecatrienoic acid—while 11 eicosanoids were decreased compared to that in healthy controls. These decreases included DHA, lyso-platelet activating factor, 12-HETE, dihydroxy-eicosatrienoic acids, dihydroxy-octadecenoic acids, 17,18-diHETE, and hydroperoxy-octadecadienoic acids (Zhang J. et al., 2021). However, in another human study, it was found that the concentrations of HETEs 19-HETE and 12-HETE were higher, while those of AA and AA derivatives, such as AEA and LTE4, were lower in patients with Parkinson’s disease (Chistyakov et al., 2024). Evaluating the clinical presentation of MS, several AA derivatives were shown to be increased. Specifically, there was a correlation between 15-hydroxyeicosatetraenoic acid and biochemical and clinical markers. Furthermore, lesser quantities of deep gray matter and the entire brain area were associated with greater 15-HETE levels (Broos et al., 2023).

Regaining tissue homeostasis requires the resolution of inflammation. When resolution is unsuccessful, pro-inflammatory cytokines and mediators are released in excess, which can lead to persistent neuroinflammation and neurodegeneration. Therefore, the production of certain SPMs is essential for both preventing and treating neurodegeneration (De Wit et al., 2021). By modifying the pro-inflammatory gene expression, influencing macrophage activity, and encouraging the resolution of neuroinflammation, SPMs have enormous potential for neuroprotection in Alzheimer’s disease. In Parkinson’s disease, SPMs can cross the blood–brain barrier, suppress microglial activation, and reduce inflammation-related markers, likely through their capacity to downregulate the NFκB signaling pathways (Ponce et al., 2022). SPMs are also out of balance in MS. SPMs are essential for maintaining the balance between effector and regulatory T-cells, and it is well known that they can regulate the mechanisms leading to chronic inflammation in MS patients (Zahoor and Giri, 2021).

ECS exerts broad neuroprotective and immunomodulatory effects in neurodegenerative diseases by reducing neuroinflammation, oxidative stress, excitotoxicity, and apoptosis. CB2 activation in microglia and the infiltrating immune cells helps regulate immune responses. Endocannabinoids also support the blood–brain barrier integrity and contribute to the clearance of toxic molecules such as Aβ (Vasincu et al., 2022). However, brain pathology in neurodegenerative diseases characterized by myelin or oligodendrocyte abnormalities, neurofibrillary tangles, and Aβ plaques is closely linked to disrupted endocannabinoid signaling. Elevated CB2 expression in microglia and astrocytes is a common feature of Parkinson’s disease, Huntington’s disease, Alzheimer’s disease, amyotrophic lateral sclerosis, and MS. In addition, reactive astrocytes in Alzheimer’s disease and MS show increased AEA hydrolysis activity, while upregulated diacylglycerol lipase β in microglia promotes 2-AG-derived PG production (Bernal‐Chico et al., 2023).

### 6.5 Cancer

Cancer is currently one of the leading causes of death in most countries worldwide. Due to the considerable influence of demographic changes, such as population growth and aging, on the divergent patterns in cancer incidence in different locations, it is anticipated that the number of cancer patients worldwide would increase during the next 50 years (Soerjomataram and Bray, 2021).

#### 6.5.1 Microbiota-derived lipid metabolites and cancer

SCFAs exert anti-inflammatory effects by inhibiting NF-κB signaling, reducing the release of pro-inflammatory cytokines such as TNF-α, increasing anti-inflammatory factors including IL-10 and TGF-β, and supporting the differentiation of naïve T-cells into Tregs that help control immune responses. They also exert epigenetic effects by inhibiting specific histone acetyltransferases, thereby influencing the expression of numerous genes and signaling pathways implicated in cancer development. SCFAs can inhibit the growth of cancerous stem cells, potentially slowing or preventing tumor initiation or recurrence by targeting mutated oncogenic pathways and by enhancing the tumor-suppressor expression (Feitelson et al., 2023). Moreover, SCFAs can have an impact on apoptosis and cell-cycle control. In MCF-7 cells, GPR41/43 activation increases intracellular Ca^2+^ levels and encourages MAPK p38. These findings are closely related to cellular stress reactions and potentially the development of cancer. Additionally, GPR43 is absent from colon cancers and metastatic cells, indicating its involvement in carcinogenesis. Following the restoration of GPR43 expression in adenocarcinoma cell lines, G0/G1 cell-cycle arrest and apoptosis have been observed (Mirzaei et al., 2021).

There is growing evidence that SCFAs may also contribute to the effectiveness of chemotherapy, immunotherapy, and radiation therapy. SCFAs can boost intra-tumoral HDACs, T-cells, and TNF-α during chemotherapy, thus increasing chemosensitivity and tumor death and preventing cell migration, invasion, and proliferation. SCFAs can improve the anti-tumor immune response and reduce the development of tumors by boosting intra-tumoral T-lymphocytes, INF-γ, and TNF-α during immunotherapy. Higher amounts of fecal SCFAs and butyrate-producing bacteria are also associated with a greater response to radiation (Al-Qadami et al., 2022).

In the context of other microbiota-derived lipid metabolites, secondary BAs can activate different signaling pathways and regulate several physiological and pathological processes. They can then impede the apoptosis of cancer cells, accelerate the cancer cell cycle, increase the capacity of cancer cells to metastasize, and promote the transformation of cells into cancer stem-cells. Secondary BAs can also indirectly encourage cancer by controlling immune cell activity (Yang and Qian, 2022). Furthermore, because of their cytotoxicity, BAs often play a role in increasing cancer angiogenesis by attracting angiogenic precursor cells and encouraging the release of pro-angiogenic proteins (Fu et al., 2022). However, according to Qi and colleagues, activation of TGR5 by BAs and TGR5-dependent signaling pathways can prevent the proliferation and migration of liver, gastric, colorectal, and breast cancers while also promoting the development and migration of lung, endometrial, and pancreatic cancers. As a result, TGR5 plays two roles in the development of cancer (Qi et al., 2022). This may be explained by differences in BA receptor and transporter expression, along with cell-specific variability in the responses triggered by receptor activation. The concentrations utilized in the experiments also affect how BAs affect neoplasias. While BAs have anti-cancer benefits in some models at low concentrations, they have pro-cancer effects at super-physiological quantities. This phenomenon is associated with their amphipathic properties and the activation of additional off-target pathways that are not engaged under physiological concentrations (Režen et al., 2022).

#### 6.5.2 Cell-component-derived structural lipids and cancer

Inflammation is widely recognized as a central factor in the development and progression of many different types of cancers, and LPS is one of the bacterial cell components that stimulate inflammation. LPS can engage the NF-κB pathway *via* TLR-4 to enhance the inflammatory response, which aggravates gut barrier failure and supports colorectal cancer growth. A healthy and intact gut barrier blocks antigens and microorganisms from crossing the intestinal epithelial layer and entering the bloodstream. On the other hand, a compromised intestinal barrier causes inflammatory reactions and increases the risk of cancer (Li Q. et al., 2023). The effect of LPS on inflammation and immune response can trigger the formation of other types of cancer along with colon cancer. A study showed that through an immunosuppressive microenvironment marked by the accumulation of myeloid-derived suppressive cells and regulatory T-cells, LPS-mediated chronic inflammation increased the programmed cell death-1 (PD-1)/programmed cell death ligand-1 (PD-L1) axis, induced T-cell exhaustion, and increased lung tumorigenesis in mice (Liu et al., 2021). Similarly, LPS contributes to the growth of hepatocellular carcinoma (Peng et al., 2022) and pancreatic cancer (Yin et al., 2021) by stimulating PD-L1 expression and successfully increasing the resistance of cancer cells to T-cell cytotoxicity.

The development, differentiation, aging, and death of cancer cells are intimately associated with biologically active sphingolipids. Certain sphingolipids, such as ceramides, are advantageous metabolites in the sphingolipid metabolic pathway that often mediate antiproliferative responses by preventing the growth and migration of cancer cells and by initiating apoptosis and autophagy. On the other hand, other sphingolipids, such S1P, have the reverse effect, causing cancer cells to change, migrate, and proliferate (Li et al., 2022). One explanation could be because S1P signaling inhibits apoptosis while promoting neovascularization and proliferation in addition to its pro-inflammatory action. Thus, it has been demonstrated that a continuous activation of STAT3 in cancer cells is closely related to S1P/S1P receptor 1 signaling. A transcription factor for the S1pr1 gene, STAT3 is activated by increased S1pr1 expression, which also increases the expression of the IL-6 gene and the activity of Janus kinase 2 (JAK2) tyrosine kinase. Aberrant IL-6–JAK–STAT3 signaling has a major impact on how cancer develops, begins, and progresses (Piazzesi et al., 2021). Drug resistance linked to cancer may also be caused by sphingolipid trafficking and deregulation of its metabolism. The ABCB1 gene, which causes drug efflux from cancer cells, is increased because of elevated levels of the enzyme glucosylceramide synthase, which is involved in sphingolipid metabolism. Drugs that cause tumor cells to undergo apoptosis are rendered useless by these bypass mechanisms (Zhakupova et al., 2025).

Through several interrelated pathways, phospholipid metabolism is also essential for immune evasion, tumor growth, and treatment resistance. For example, lysophosphatidic acid can activate GPCRs to enhance cancer cell proliferation, invasion, metastasis, and resistance to chemotherapy. It also reshapes the tumor microenvironment by promoting angiogenesis and fibrosis, driving macrophages toward an M2 phenotype, and suppressing cytotoxic T-cell activity (Delmas et al., 2025; Aiello and Casiraghi, 2021). Additionally, it was reported that lysophosphatidic acid, along with its receptors, are associated with cancer progression. Large-scale transcriptomic analysis of independent breast cancer patient cohorts demonstrates that high expression of lysophosphatidic acid receptors 1, 4, and 6 in breast tumors is associated with a less aggressive phenotype, whereas high expression of lysophosphatidic acid receptor 2 is associated with higher tumor grade, greater mutational burden, and poorer survival (Benesch et al., 2023).

#### 6.5.3 Lipid mediators and cancer

Pro-inflammatory eicosanoids, which are produced by both tumor cells and stroma, can affect tumor growth through several pathways. These physiologically active lipids have the ability to affect the invasion, migration, apoptosis, and proliferation of tumor cells. In addition, they can shape the tumor microenvironment by influencing tumor neo-angiogenesis and tumor antigenicity (Patrignani et al., 2023). For example, the elevation of COX-2 expression and the following increase in PGE2 levels can trigger and promote hepatocellular carcinoma development by activating multiple signaling pathways, such as Akt, STAT3, and vascular endothelial growth factor (VEGF). LTB4 were also engaged in lung metastasis from hepatocellular carcinoma cells (Alvarez and Lorenzetti, 2021). Dysregulation of eicosanoids has been linked in the formation and progression of brain malignancies, including glioblastoma, meningioma, and medulloblastoma (Chen et al., 2023). In a clinical study, tetranorPGJM (metabolite of PGD2) and 11-Dehidro TXB2 were significantly elevated in non-small-cell lung cancer patients than in control. These changes reflected systemic dysregulation tied to mast cell and platelet activation, and the LTE4 levels were higher in advanced non-small-cell lung cancer and distinguished between the early and advanced stages of the disease (Zelkowska et al., 2025). Complementing these human findings with *in vivo* experimental data, another study demonstrated that PGD2, prostacyclin, and TX levels are elevated in mouse models of neoplasia, while PGE2, 12-hydroxyheptadecatrienoic acid, HETEs, and hydroxylated DHA metabolites are particularly elevated in pancreatic tumors. In patient samples, a switch from PGD2 to PGE2-producing enzymes has been reported in the epithelium during the transition to pancreatic ductal adenocarcinoma (Gubbala et al., 2022). Providing direct translational evidence from patient tissue samples, in another study, it was demonstrated that colorectal cancer tissues exhibit a pronounced pro-inflammatory lipid profile characterized by increased arachidonic acid-derived mediators and a deficiency of pro-resolving lipid mediators such as lipoxins. By integrating lipidomics with transcriptomic analyses, the authors showed that defective lipid class switching may contribute to persistent tumor-associated inflammation and colorectal cancer progression (Soundararajan et al., 2025).

As opposed to pro-inflammatory eicosanoids, the anti-tumor immunity may be enhanced by SPMs. Lipoxin A4 and RvD1 suppress angiogenesis by inhibiting VEGF and acting on cancer cell surface receptors to block the key steps in invasion and metastasis. RvD2 promotes M2 macrophage phagocytosis, infiltration, proliferation, and survival, while also reducing the release of pro-inflammatory cytokines. Additionally, lipoxin A4 inhibits tumor-promoting Breg cells and enhances the anti-tumor activity of immune cells such as Tregs and neutrophils (Torres et al., 2023). Furthermore, inflammation can be caused by cancer therapies such as chemotherapy, radiation, immunotherapy, or surgery. Tumor debris produced by cell death through these treatments attracts pro-inflammatory neutrophils and macrophages, which produce cytokines and eicosanoids. However, SPMs promote the removal of tumor debris and help counterbalance the eicosanoid-driven cytokine storms (Quinlivan et al., 2025).

ECS can influence multiple stages of tumor development. Cannabinoids stimulate apoptosis and autophagy; trigger cell-cycle arrest; and suppress tumor cell migration, invasion, and self-renewal. Therefore, ECS dysregulation has been linked to carcinogenesis and may be the cause of cancer aggressiveness (Braile et al., 2021). Cannabinoids can inhibit cell-cycle progression (by decreasing cAMP levels, increasing ROS levels, or inhibiting the PI3K/Akt pathway), induce apoptosis (by increasing ROS levels, increasing caspases levels, or inhibiting the PI3K/Akt or MAPK pathways), and induce autophagy (by increasing ceramide levels or increasing endoplasmic reticulum stress) (Pagano et al., 2021). *In vitro* evaluation of chemoresistant ovarian cancer cell lines demonstrated that when AEA and 2-AG were administered together with chemotherapy drugs in ovarian cancer patients, an increase in endoplasmic reticulum stress and autophagy and a decrease in chemoresistance were reported in chemotherapy-resistant cancer cells (Lin et al., 2023). By modulating the functionality and reactivity of distinct cellular components, ECS also re-shape the tumor microenvironment. Several tumor microenvironment cells express receptors of the ECS: specifically, immune cells, cancer-associated fibroblasts, and endothelial cells. Cannabinoids can affect the immunological components by decreasing cytokine release, T-cell recruitment, proliferation, and M2 population rate. They can also lessen the invasiveness and reactivity of cancer-associated fibroblasts. Cannabinoids affect the migratory, invasion, and sprouting characteristics of endothelial cells and decrease the release of angiogenic factors (Iozzo et al., 2021). Moreover, cannabinoids provide a range of palliative benefits for cancer patients, including the reduction of nausea and vomiting, appetite stimulation, analgesic effects, mood enhancement, and improvement of sleep disturbances (Braile et al., 2021).

### 6.6 Chronic kidney disease

Kidney diseases are currently the seventh most important risk factor for death worldwide, and their incidence is increasing. Moreover, demographic trends, the obesity pandemic, and the sequelae of climate change are all projected to increase the prevalence kidney disease further, with major implications for survival, quality of life, and healthcare spending worldwide (Francis et al., 2024). Kidney diseases share immune dysregulation and metabolic disturbances, where immune cells, cytokines, and complementary factors drive inflammation and fibrosis. Oxidative stress, altered glucose and lipid metabolism, and mitochondrial dysfunction can also exacerbate renal injury and disease progression (Qu and Jiao, 2023).

#### 6.6.1 Microbiota-derived lipid metabolites and chronic kidney disease

The physiological functions of SCFAs in slowing the progression of chronic kidney disease are gradually being understood. These functions include enhanced energy metabolism, immunological pathways, oxidative stress suppression, autophagy modulation, and inhibition of inflammatory reactions. However, it was demonstrated that there was a considerable decline in the bacterial community that produces SCFAs in chronic kidney disease. As the renal function deteriorates, this loss in flora abundance becomes more pronounced, and the degree of this decline shows a positive link with the disease’s course (He et al., 2024). A related human study found that fecal propionate and butyrate levels in chronic kidney disease patients gradually decreased as the disease progressed and correlated closely with clinical parameters such as estimated glomerular filtration rate, serum creatinine, and blood urea nitrogen (Corte-Iglesias et al., 2024). A systematic review that included 25 studies with a total of 1,436 CKD patients and 918 healthy controls also demonstrated changes in the gut microbiome (phylums *Proteobacteria* and *Fusobacteria* are more abundant, while *Roseburia, Faecalibacterium, Pyramidobacter,* Prevotellaceae*_UCG-001*, and *Prevotella_9* are less abundant) and a decrease in the concentration of SCFAs (Zhao et al., 2021). Furthermore, the wide range of comorbidities associated with chronic kidney disease—including dysfunctions of the cardiovascular, pulmonary, ocular, central nervous, musculoskeletal, gastrointestinal, mitochondrial, and immune systems—arise from sustained systemic inflammation, oxidative stress, and the accumulation of toxic metabolites. However, it is hypothesized that gut microbiota may ameliorate kidney damage and complications by regulating the inflammatory and immune responses (Magliocca et al., 2022). By inhibiting HDAC and activating GPCRs, SCFAs promote Treg differentiation in the gut. When HDACs are inhibited, the mucosal immune system’s NF-κB is suppressed, which affects the transcription of genes linked to inflammation, such as TNF-α and IL-6. Furthermore, GPCR activation may influence inflammatory processes in part by reducing NF-κB expression and increasing colonic Treg differentiation (Tsuji et al., 2025). The nuclear protein Nrf2 is also directly activated and ROS are produced when SCFAs bind to receptors (Magliocca et al., 2022).

Another metabolite affected by dysbiosis in microbiota is BAs. Especially, *Eggerthella lenta* was found to be positively connected with higher levels of secondary BAs in chronic kidney disease patients when compared to the control group in an observational study (Wang et al., 2020). In another clinical case-control study, microbial genes associated with biosynthesis of secondary BAs were reported to be differentially abundant in early chronic renal failure patients, and a positive correlation was detected between *Clostridium glycyrrhizinilyticum* and glycoursodeoxycholic acid, a secondary BA (Wu et al., 2020). However, on evaluating more advanced clinical stages, plasma secondary BA levels were found to be significantly lower in patients with end-stage chronic kidney disease undergoing hemodialysis than in controls in a study. This was explained by the fact that a decrease in salt hydrolase enzyme, even due to disruption in the gut microbiota, may prevent the production of secondary BAs (Li et al., 2019). Disturbances in BA metabolism can lead to defective energy homeostasis and immune dysregulation by altering glucose and lipid metabolism and impairing immune cell functions, including macrophage polarization and the differentiation of Th17 and Treg cells. Kidney disease, eventually, can progress more quickly as a result of these impacts (Xu et al., 2024).

#### 6.6.2 Cell-component-derived structural lipids and chronic kidney disease

Podocytes, the glomerular basement membrane, and glomerular endothelial cells make up the glomerular filtration barrier. For the glomerular filtration barrier to continue operating properly, podocyte integrity is essential. Proteinuria is the term for the leaking of proteins into urine caused by podocyte malfunction and the loss from the glomerular filtration barrier as a result of cellular stress, genetic mutations, inflammation, or lipotoxicity (Mallela et al., 2022). While the exact mechanism by which abnormalities in sphingolipid metabolism lead to podocyte damage remains unclear, several diseases highlight the critical role that sphingolipids play in preserving podocyte health. Alteration in sphingolipid metabolism may also be associated with chronic renal failure *via* ROS production, the renin–angiotensin–aldosterone system, renal fibrosis, and inflammation (Šakić et al., 2024). Moreover, S1P can exert pro- or anti-fibrotic actions in the kidney depending on where it is located and which S1P receptor subtype it engages. Spinster-2 transports intracellular S1P to the extracellular space. Once outside the cell, S1P can activate TGF-β pathways, triggering Smad2/3 phosphorylation and promoting extracellular matrix accumulation, thereby accelerating renal fibrosis. In contrast, intracellular S1P helps limit fibrosis, and S1P receptor 1 activation in tubular epithelial cells is protective. S1P is produced by the enzymes sphingosine kinases (SphK1 and SphK2), which have opposing roles: SphK1 counters fibrosis, while SphK2 promotes it (Schwalm et al., 2024).

Lysophosphatidylcholine accumulated excessively because of abnormal lipid metabolism in the kidney, which also led to organelle stress and apoptosis. In the study conducted in rats, the lysophosphatidylcholine (16:0) and (18:0) levels in urine and renal tubulointerstitium were shown to increase as the disease progressed (Yoshioka et al., 2022). In another study, it was found that renal lysophosphatidic acid levels increase in several kidney disease rodent models, including hypertension, type 1 and type 2 diabetes, and obstructive nephropathy (Hirata et al., 2021). *In vitro* evaluation of human proximal tubular epithelial cells demonstrates that lysophosphatidic acid responses cluster with pro-inflammatory stimuli such as TNF and IL-1, activating numerous inflammation-related signaling hubs while concurrently increasing the secretion of clinically important inflammatory mediators (Magkrioti et al., 2022).

#### 6.6.3 Lipid mediators and chronic kidney disease

Several oxylipins are produced by omega-6 and omega-3 long-chain PUFAs, which influence both pro-inflammatory and anti-inflammatory autocrine and paracrine signaling in chronic kidney disease. For example, PGD2 exerts renoprotective effects through the inhibition of the PPAR-γ pathway and the release of pro-inflammatory cytokines and antifibrotic effects through the inhibition of TGFβ (Sharma et al., 2022). However, TXA2 levels are increased in chronic kidney disease. Increased TXA2 levels may increase ROS production, thrombotic events, and endothelial dysfunction, leading to cardiovascular complications in kidney patients (Fijałkowski et al., 2018). Eicosanoid metabolites such as 20-Oxo-LTE4, LTE3, and 20-Oxo-LTB4 were also shown to be considerably elevated in rats with chronic renal damage. The serum creatinine levels and these three eicosanoids showed a high correlation, which may indicate renal impairment (Wang et al., 2021). Additionally, longitudinal data from a well-characterized human clinical cohort study demonstrated that a considerably higher level of 20-HETE was independently linked to end-stage kidney disease (Afshinnia et al., 2018).

Persistent low-grade inflammation can contribute to the development of chronic renal failure through ongoing interactions between resident and/or circulating immune cells and parenchymal cells. However, SPMs can prevent deterioration in renal function. Lipoxins reduce fibrosis attenuation, collagen deposition, and albuminuria and increase macrophage efferocytosis and IL-10 production. Rvs reduce leukocyte infiltration, fibroblast proliferation, and collagen deposition and increase Treg. Maresins and protectins protect kidney function by reducing ROS production and inhibiting NF-κB activity (Brennan et al., 2021; Rispoli et al., 2025). Additionally, SPMs stimulate macrophage-driven efferocytosis of apoptotic cells, prevent neutrophil infiltration and degranulation, and trigger neutrophil programmed cell death. Thus, it prevents persistent neutrophil activation, which is recognized as a key factor in the progression of kidney disease (Kang et al., 2025). However, a clinical study conducted on patients receiving hemodialysis showed a decrease in plasma EPA and DHA-derived oxylipin metabolite levels compared to that in the control group (Watkins et al., 2022).

ECS, particularly its receptor CB1, has effects on kidney function. Activation of the CB1 receptor normally promotes the movement of ions and proteins in various nephron compartments and controls renal vascular hemodynamics. However, cannabinoids produced in various kidney cells due to obesity and diabetes activates CB1 receptors, thereby promoting oxidative stress, inflammation, and the development of kidney fibrosis (Tam, 2016). Diabetic nephropathy was brought on by the over-activation of CB1, which damaged podocytes because of elevated renin–angiotensin–aldosterone activity, albuminuria, and hyperglycemia. Additionally, CB1 increases the mammalian target of rapamycin complex 1 (mTORC1) and GLUT-2 activity in renal proximal tubule cells of patients with diabetic nephropathy (Arceri et al., 2023). Therefore, CB1-inhibition can directly prevent kidney fibrosis in metabolic and non-metabolic nephropathies (Dao and François, 2021).

## 7 Therapeutic opportunities

### 7.1 Modulating microbiota to influence lipid mediator profiles

The gut microbiota is capable of both converting and synthesizing lipids. It can also break down dietary lipids, producing products with these regulatory properties (Brown et al., 2023). The gut microbiota has emerged as a key regulator of these lipid metabolites and influences inflammatory processes. Indeed, dysbiosis can lead to alterations in lipid metabolic pathways, thereby influencing lipid mediator profiles (Ávila-Román et al., 2021; Chattopadhyay et al., 2022). In an experimental study supporting this relationship, male Wistar rats were fed either a standard diet or a cafeteria diet for 5 weeks; in the last 2 weeks, a cocktail of antibiotics (ampicillin, vancomycin, and imipenem) was administered to induce gut microbiota dysbiosis. Both the cafeteria diet and antibiotic administration significantly altered the plasma oxylipin (OXL) profiles. Additionally, a positive correlation was demonstrated between *Proteobacteria* and the pro-inflammatory OXL LTB_4_. These results indicate that the gut microbiota plays a role in OXL metabolism (Ávila-Román et al., 2021).

The gut microbiota may shape lipid mediator profiles by regulating the bioavailability of fatty acid precursors involved in lipid mediator synthesis. This is because the gut microbiome is found throughout the entire digestive system of mammals and encounters all dietary lipids entering the body (Brown et al., 2023). Using a murine model, researchers showed that gut microbial communities mediate the conversion of fatty acid epoxides to fatty acid diols in the colon, thereby regulating colonic concentrations of these lipid mediators and their associated biological effects (Jing et al., 2025). Additionally, the microbiota influences the digestion, absorption, and metabolism of PUFA such as omega-6 and omega-3, obtained through diet (Kumar et al., 2025). Enzymes in the gut microbiome act like a second liver, breaking down, transforming, and detoxifying dietary components. This can have beneficial or harmful effects on the host’s health. For example, dietary omega-6 PUFAs (such as LA) are converted into hydroxy fatty acids (HYA, HYB, and HYC) by microbial enzymes derived from *Bifidobacterium* and *Lactobacillus* species; these metabolites have been reported to play a role in the regulation of glucose homeostasis and inflammatory responses *via* GPR40 and GPR120 receptors. Omega-3 PUFAs (such as ALA) are converted into conjugated linolenic acids by the gut microbiota, and this conversion is associated with *Bifidobacterium*, *Lactobacillus*, and *Streptococcus* species. The metabolites thus formed have been shown to be associated with suppressing inflammation, limiting oxidative stress, and inhibiting pathogenic bacteria (Brown et al., 2023).

On the other hand, the gut microbiota plays crucial roles in BA-related physiological processes (Zhao M. et al., 2025) by participating in biotransformation processes such as deconjugation, dehydroxylation, and reconjugation of BAs (Ramírez-Pérez et al., 2018). Once primary BAs enter the gastrointestinal system, they are converted into secondary BAs by the gut microbiota (Winston and Theriot, 2020). In addition, the gut microbiota influences the composition of the BA pool. These biotransformations modulate the signaling properties of BAs through the TGR5 and the FXR, which regulate various metabolic pathways in the host, thereby contributing to the regulation of lipid and glucose metabolism (Ramírez-Pérez et al., 2018). Supporting this mechanism, individuals with intrahepatic cholestasis of pregnancy have altered gut microbiota composition, particularly an increase in *Bacteroides fragilis*, which impairs BA synthesis and excretion by suppressing FXR signaling *via* BSH activity (Tang et al., 2023).

SCFAs derived from the gut microbiota *via* the fermentation of partly digestible and indigestible polysaccharides (Jian et al., 2014) exert effects on lipid metabolism (Campos-Perez and Martinez-Lopez, 2021) and have been reported to indirectly influence the production of inflammatory lipid mediators (Soliman et al., 2013). SCFAs regulate the function and size of the colonic Treg pool. Tregs, in turn, are critical in regulating intestinal inflammation (Smith et al., 2013).

SCFAs are highly effective in cellular signaling pathways by activating GPCRs (GPR41 and GPR43) and inhibiting HDAC. SCFAs also modulate macrophage production of inflammatory mediators. Specifically, butyrate suppresses the LPS and cytokine-induced production of the pro-inflammatory mediators nitric oxide (NO), IL-6, and TNF-α (Vinolo et al., 2011). These signaling pathways can influence eicosanoid production by altering NF-κB and COX expression (Kovarik et al., 2013; Tayyeb et al., 2020). In an experimental study, PGE_2_ levels measured in primary astrocytes following stimulation with LPS were reduced to the control levels by acetate administration (Soliman et al., 2013). In a study conducted in human monocytes, the bacterial fermentation product n-butyrate was observed to markedly induce PGE_2_ production by increasing PTGS2 (COX-2) expression in monocytes following the activation of TLR 2 and TLR 4, while also inducing the secretion of LTB_4_ and TXB_2_. However, it has been reported that the NF-κB signal is affected differently depending on the phase (Kovarik et al., 2013). Therefore, conflicting effects regarding the role of PGE_2_ in inflammation have been reported in the literature (Vinolo et al., 2011; Martín-Vázquez et al., 2023). PGE_2_ is described both as a driver of inflammation and has been associated with the restoration of tissue homeostasis under stress or pro-inflammatory conditions. These effects are reported to vary depending on the local inflammatory context and PGE_2_ levels (Martín-Vázquez et al., 2023). In this way, SCFAs regulate various leukocyte functions, including the production of eicosanoids, chemokines such as MCP-1 and cytokine-induced neutrophil chemoattractant-2 (CINC-2), along with cytokines such as IL-2, TNF-α, IL-10, and IL-6, thereby regulating inflammatory processes (Vinolo et al., 2011).

The gut microbiota is not only effective in suppressing pro-inflammatory lipid mediators but has also been reported to support the biosynthesis of lipid mediators involved in resolving inflammation, particularly through specific bacterial strains (Speckmann et al., 2025). The resolution process of inflammation is characterized by lipid mediator class switching along with temporal regulation of leukocyte migration. In this process, signals from the initially produced pro-inflammatory lipid mediators trigger the synthesis of lipid mediators that support the resolution of inflammation (Spite et al., 2014). Indeed, lipid mediator class switching is considered a critical mechanism for the controlled termination of the inflammatory response. Pro-resolution lipid mediators such as maresins, protectins, and Rvs are critical in terminating the inflammatory response and restoring tissue homeostasis (Serhan et al., 2015).

### 7.2 Biotics in lipid-related therapy

With an improved understanding of the regulatory role of the gut microbiota in lipid mediator biology (Jing et al., 2025; Ávila-Román et al., 2021), probiotics, prebiotics, and post-biotics are also considered to represent potential therapeutic approaches in this context. These probiotics, prebiotics, and post-biotics modulate various mechanisms, including promoting the growth of beneficial microbes, SCFA production, regulating immune responses, strengthening the gut barrier, and regulating immune responses, and they also exert an effect on the host’s lipid metabolism. In this way, they can indirectly but effectively influence the lipid mediator profile (Smolinska et al., 2025).

Probiotics are live microorganisms that provide health benefits to the host when administered in sufficient quantities (Salminen et al., 2021) and exert effects on lipid metabolism through various mechanisms. SCFAs can be produced by fermenting dietary fiber by probiotic microorganisms. Furthermore, primary BAs are converted into secondary BAs by the action of probiotics. The FXR signaling pathway is also activated by secondary BAs. This also helps to decrease low-density lipoprotein production and accelerate the breakdown of lipids. Through these mechanisms, probiotics exert a regulatory effect on multiple steps of lipid metabolism (Song et al., 2023). In an *in vitro* study supporting these mechanisms, VSL#3 probiotics (a proprietary probiotic mixture containing *Lactobacillus acidophilus*, *Lactobacillus bulgaricus*, *Lactobacillus casei*, *Lactobacillus plantarum*, *Bifidobacterium breve*, *Bifidobacterium infantis*, *Bifidobacterium longum*, and *Streptococcus salivarius subsp. thermophilus*) were shown to promote subsequent fecal BA excretion and ileal BA deconjugation and to induce hepatic BA neosynthesis through the downregulation of the gut–hepatic FXR–FGF15 axis (Degirolamo et al., 2014). In a clinical study, the use of BSH-active *Lactobacillus reuteri* NCIMB 30242 was shown to rapidly and significantly alter BA metabolism in humans. The delayed-release form increased the total, conjugated, and deconjugated BAs, particularly in the first week. These increases were found to be positively correlated with FGF-19 and negatively correlated with plant sterols. The findings showed that, in addition to its cholesterol-lowering effects, the BSH-active probiotic *Lactobacillus reuteri* NCIMB 30242 induced significant increases in the total, conjugated, and unconjugated BAs, indicating that it can also exert a significant and rapid effect on BAs metabolism (Martoni et al., 2015).

It has been reported that probiotics also improve the intestinal barrier function, reduce LPS translocation, suppress TLR-mediated inflammatory signaling, decrease inflammatory cytokine production, and modulate metabolic signaling pathways related to lipid and eicosanoid metabolism. Probiotics can lead to changes in the production of some lipid mediators by regulating the activity of COX pathways (Plaza-Diaz et al., 2019). In a study conducted in rats, oral administration of *Bifidobacterium bifidum* was shown to activate TLR-2 in the intestinal epithelium in a rat necrotizing enterocolitis model. Additionally, it was reported to increase COX-2 expression, leading to higher PGE_2_ production in the ileum and providing protection against NEC-associated intestinal apoptosis (Khailova et al., 2010). In another study, the effect of *Lactobacillus rhamnosus GG* (LbGG), one of the most commonly used probiotics associated with colonization of the colonic mucosa, on PGE_2_ production was analyzed through the modulation of the eicosanoid pathways in the colonic mucosa. In Caco-2 cells, exposure to LbGG was found to lead to mobilization of AA, accompanied by a concurrent increase in the components of the LT and COX-2-dependent PGE2 pathways. The researchers reported that LbGG increased COX-2-mediated PGE2 secretion and contributed to the maintenance of colonic mucosal homeostasis (Uribe et al., 2018).

Prebiotics are a substrate that is selectively utilized by host microorganisms and provides health benefits (Salminen et al., 2021). They increase SCFA production by promoting the growth of *Bifidobacterium* and *Lactobacillus* species. Galactose-containing galactooligosaccharides (GOS) and fructose-containing fructans (fructooligosaccharides [FOS] and inulin-type fructans [ITF]) are among the major prebiotic sources (Smolinska et al., 2025). SCFA, formed by the fermentation of dietary fiber, has significant impacts on both lipid metabolism and the regulation of inflammatory responses (Koh et al., 2016). In Wistar–Kyoto rats fed a high-fat (HF) diet, a 10-week inulin intervention reduced the hepatic levels of certain eicosanoids, such as 11(12)-EET and 15-HETrE, and decreased systemic oxidative stress compared with the effects of the high-fat diet (Miralles-Pérez et al., 2022). Early-life supplementation with prebiotic oligosaccharides (GOS + polydextrose) increased SCFA production by altering the intestinal microbiota composition in piglets and elevated the LA and AA content in the colonic mucosa (Eudy et al., 2023). In this way, prebiotics can modulate lipid mediator synthesis and the fatty acid pool required for its synthesis *via* the microbiota and SCFA.

Post-biotics, a new type of biotic, have great potential in improving health (Smolinska et al., 2025). According to the International Scientific Association of Probiotics and Prebiotics (ISAPP), post-biotics are preparations of inanimate microorganisms and/or their components that provide health benefits to the host. The inactivated version does not have to be derived from a probiotic to be considered post-biotic. Since the microorganisms in post-biotics have lost their ability to multiply, there is no risk of bacteremia or fungemia, which are rarely reported with the use of probiotic. Therefore, post-biotics offer a safer profile compared to probiotics (Salminen et al., 2021). In recent years, post-biotics have gained increasing attention for the treatment of certain metabolic conditions due to their effects on reducing inflammation and oxidative stress, modulating the microbiota, regulating intestinal barrier function, and influencing lipid metabolism (Yilmaz, 2024; Zhou et al., 2006). It has been reported that post-biotics, particularly SCFAs, BAs, and exopolysaccharides, can exert direct or indirect regulatory effects on lipid metabolism (Salminen et al., 2021; Sanhueza-Carrera et al., 2025). With these characteristics, post-biotics can be considered promising complementary therapeutic agents for controlling lipid mediator-driven inflammation and supporting metabolic homeostasis.

### 7.3 Targeting lipid mediator pathways in microbiota-driven diseases

Over time, there has been growing evidence of a close relationship between changes in the gut microbiota and inflammatory and metabolic processes. With the relationship between the gut microbiota and lipid mediators now better understood, targeting lipid mediator pathways in therapy may represent a promising approach for the management of microbiota-associated diseases (Baptista et al., 2020). In this regard, the gut microbiota impacts the host lipid metabolism, and microbiota-induced changes can indirectly influence inflammatory processes (Brown et al., 2023; Baptista et al., 2020).

It is widely accepted that changes in the amounts and types of dietary fats can increase systemic inflammation. In addition, it has been shown that lipids directly synthesized by the mammalian microbiota can strongly stimulate mucosal and systemic immune responses. In this way, initiated inflammation has been reported to affect lipid absorption and metabolism *via* feedback mechanisms (Brown et al., 2023).

In a study analyzing the protective role of RvD2, an omega-3 fatty acid derivative, in IBDs, an exacerbated inflammatory response was observed in experimental colitis models. Micelara RvD2 application has been reported to be effective in improving the clinical condition of animals and reducing inflammation, similar to what was observed with anti-TNF-α treatment, thus demonstrating a critical immunomodulatory effect (Chaim et al., 2024). Similarly, the anti-inflammatory effects of D-series Rvs derived from DHA, which is found in fish oils, have been analyzed in experimental colitis models. The effects of aspirin-triggered RvD1 (AT-RvD1), its precursor 17(R)-hydroxy-DHA (17R-HDHA), and RvD2 were evaluated in colitis models. Treatment with 17R-HDHA, AT-RvD1, and RvD2 decreased the disease activity index and markedly decreased colonic damage and neutrophil infiltration in the colitis model. These treatments also reduced the colonic levels of the cytokines IL-1β, TNF-α, CXCL1/KC, and MIP-2, along with the mRNA expression of NF-κB and the adhesion molecules vascular cell adhesion molecule-1 (VCAM-1), lymphocyte function-associated antigen-1 (LFA-1), and intercellular adhesion molecule-1 (ICAM-1) (Bento et al., 2011). In addition to Rvs, maresin-1, a DHA derivative, also appears to be effective in diseases accompanied by inflammation (Saito-Sasaki et al., 2022). In a study analyzing the effects of maresin-1 (MaR1) in colitis models, systemic administration of MaR1 in mice was shown to significantly reduce colonic tissue damage. MaR1 has been found to reduce the ICAM-1 mRNA expression in colitis models. The study also reported that the favorable effects of MaR1 were linked to inhibition of the NF-κB signaling pathway (Marcon et al., 2013).

Beyond IBDs, similar mechanisms are reported to play a role in several other diseases accompanied by inflammation (Spite et al., 2014). The effects of RvD1, RvD2, and MaR1 were analyzed in LPS-stimulated primary human monocytes. These lipid mediators were shown to suppress the release of TNF, IL-1β, IL-8, and IL-12 p40, while enhancing IL-10 production (Gu et al., 2016). In an *in vivo* model of LPS-induced inflammation, RvE1 was reported to reduce inflammatory cytokine levels in C2C12 skeletal muscle myotubes and to prevent LPS-induced skeletal muscle atrophy (Baker et al., 2018). In a study evaluating the effects of the lipid mediator MaR1 in obese mice (diet-induced obese mice and genetically obese ob/ob mice), MaR1 administration in diet-induced obese mice was shown to reduce pro-inflammatory macrophage accumulation in white adipose tissue, decrease inflammatory cytokine expression, and enhance insulin sensitivity. In ob/ob mice, MaR1 treatment was reported to attenuate adipose tissue inflammation and improve insulin sensitivity (Martínez-Fernández et al., 2017). In another study conducted in diet-induced obese mice, MaR1 administration improved the inflammatory status of the colonic mucosa. Moreover, although it did not completely reverse dysbiosis, it partially compensated for obesity-induced alterations in the gut microbiota (León et al., 2020). Lipid mediator profiling of the aortas of mice fed a high-fat diet revealed an increase in the inflammatory lipid mediators PGE_2_ and LTB_4_ during advanced atherosclerosis. Concurrently, a decrease in the pro-resolving lipid mediators MaR1 and RvD2 was reported. Therapeutic administration of RvD2 and MaR1 was shown to prevent atheroprogression, which is characterized by the inhibition of necrotic core expansion and macrophage accumulation, along with increased smooth muscle cell content and fibrous cap thickness (Viola et al., 2016).

### 7.4 Multi-modal approaches: nutrition–microbiota–exercise axis

Nutrition plays a major role in shaping the function and composition of the human microbiota. In this regard, the content, quantity, and timing of nutrition are all influential. The complex interactions between nutrients and microorganisms are associated with beneficial or harmful consequences for individuals’ health (Zmora et al., 2019).

For example, while the Mediterranean diet exerts beneficial effects on many diseases due to its effects on gut microbiota composition, excessive alcohol consumption, processed foods, and food additives are associated with the development of dysbiosis and inflammatory and metabolic disorders (Zhang L. et al., 2025)

At the same time, high dietary intakes of sugar, SFAs, and excessive protein have been associated with gut inflammation and related diseases. In contrast, dietary fiber or probiotics have beneficial health effects, including increased SCFA, decreased indoxyl sulfate, and increased antioxidants. It is also associated with lower intestinal inflammation and improved lipid metabolism (Valdes et al., 2018). During an acute inflammatory response, pro-inflammatory lipid mediators such as ARA-derived PGs and LTs, which are derived from omega-6 PUFAs, are predominant. Over time, a shift in lipid mediator class occurs. The generation of pro-resolving mediators, such as E-series Rvs originating from the omega-3 fatty acid EPA, D-series Rvs, maresins, and protectins originating from DHA, along with lipoxins originating from the omega-6 fatty acid ARA, is elevated. The balance between the pro-inflammatory and pro-resolution mediators plays a decisive role in the chronicity or resolution of inflammation (Serhan et al., 2024). ARA released from membrane phospholipids is metabolized into PGs and LTs through the 5-LOX, COX-1, and COX-2 pathways. Meanwhile, EPA is involved in the synthesis of E-series Rvs *via* the 15-LOX and 5-LOX pathways, whereas DHA participates in the synthesis of D-series Rvs, maresins, and protectins through the 5-LOX, 12-LOX, and 15-LOX pathways (Biełach-Bazyluk et al., 2026).

A meta-analysis recommending omega-3 PUFAs as adjuvant anti-inflammatory agents reported that omega-3 PUFA supplementation in adults could reduce IL-6, C-reactive protein (CRP), and TNF-α concentrations (Kavyani et al., 2022). However, the membranes of cells involved in inflammation are rich in omega-6 PUFAs, particularly ARA. High omega-6 PUFA intake has been associated with inflammation due to ARA being a precursor of pro-inflammatory lipid mediators (Innes and Calder, 2018).

Although preclinical studies have indicated that SPMs derived from DPA, EPA, and DHA play regulatory roles in the active resolution of inflammation, data regarding the translation of these mechanisms to humans remain limited (Schwab et al., 2007; Serhan et al., 2009). In a study conducted in older subjects with low-level chronic inflammation (CRP>2 μg/mL), the associations between plasma omega-3 fatty acid levels, their derived SPMs, and inflammatory markers were evaluated. Phospholipid DHA content was found to be inversely correlated with IL-6, TNF-α, IL-10, and MCP-1 levels. In addition, MCP-1 was negatively related to the DHA-derived 14-HDHA and 4-HDHA; whereas IL-10 was found to be negatively related to the EPA-derived 18-HEPE, 5-HEPE, and 12-HEPE; the DPA-derived Rv5DPA; and the DHA-derived 4-HDHA. The study results support the idea that dietary omega-3 fatty acids have anti-inflammatory effects and indicate that lipid mediators derived from DPA, DHA, and EPA may be associated with the regulation of inflammatory processes in the context of chronic inflammation (Lamon-Fava, 2025).

On the other hand, exercise, as an environmental factor, plays an important role in determining changes in microbial composition (Monda et al., 2017). Exercise can have an effect on increasing the number of useful microbial species, improving the growth of commensal bacteria, enriching microflora diversity, and reducing intestinal inflammation (Monda et al., 2017; Campbell and Wisniewski, 2017). In a systematic review, higher levels of physical activity and cardiorespiratory fitness were associated with greater fecal bacterial alpha diversity and an increased representation of certain phyla and specific SCFAs (Ortiz-Alvarez et al., 2020).

The effects of physical exercise on the gut microbiota occur through multiple interrelated mechanisms. Exercise influences gut microbiota composition through intestinal immune system activity, BAs, SCFAs, and increased intestinal motility. The decrease of pro-inflammatory cytokines, together with an increase in the anti-inflammatory cytokines, contributes to an increase in Treg. Exercise, which promotes an increase in primary BAs, thereby supports an increase in CA and the *Firmicutes/Bacteroidetes* ratio. In addition, exercise can increase SCFA production, particularly butyrate levels. Increased butyrate contributes to the improvement of intestinal barrier function through the regulation of cytokine responses, Hsp70 expression, mucus synthesis, and the support of tight junction integrity (Costa et al., 2017). Butyrate supplementation and exercise substantially suppressed the relative abundance of LPS-producing phyla. The expression of CRTC2 and SESN2 in the livers of mice was markedly elevated following exercise and/or butyrate supplementation. In conclusion, it was determined that exercise improves lipid metabolism *via* the butyrate–SESN2/CRTC2 pathway (Yu C. et al., 2019).

Exercise also appears to affect intestinal permeability, with effects varying by duration and intensity. Intense and acute physical exertion generally increases intestinal permeability, whereas regular low-to-moderate-intensity exercise usually has a positive effect on intestinal integrity and reduces permeability. This situation may be linked to the chronic activation of HIF-1α, which induces the transcription of genes that enhance intestinal barrier function, and to an increase in the steady-state levels of heat shock proteins (Dmytriv et al., 2024).

Based on all of this, the impact of both diet and physical exercise on gut microbiota regulation is clear (Ge et al., 2023). However, combining diet and physical exercise offers a more effective approach to preventing metabolic diseases by altering the gut microbiota (Zhang L. et al., 2022). The gut microbiota shaped by exercise and diet contributes to reduced oxidative stress and supports the immune system through SCFAs, BAs, and tryptophan metabolites (Zhang X.e. et al., 2025). In conclusion, nutrition, physical activity, and the gut microbiota comprise interrelated processes that regulate host metabolism, immune responses, and inflammation (Campaniello et al., 2022; Fan and Pedersen, 2021).

In rats fed a standard diet, exercise significantly altered the gut microbiota composition, whereas in rats fed a high-fructose and high-fat diet, it had no significant effect. The study results show that the effects of exercise on the gut microbiota may vary depending on the diet composition (Batacan et al., 2017). In a study conducted in athletes, it was reported that the group characterized by healthy dietary habits and regular physical activity exhibited distinct metabolic separation in urinary and fecal proton nuclear magnetic resonance (^1^H-NMR) metabolomic profiles, along with significant differences in fecal SCFA levels and microbial diversity (Penney et al., 2020). Furthermore, combining diet and exercise is an effective strategy for reducing inflammatory complications (Sobreviela Sánchez et al., 2025), and reduced inflammation can also alter the lipid mediator profile (Serhan et al., 2007). In this context, the combination of diet and exercise can exert complementary effects on cytokine profiles, lipid mediator balance, and the gut microbiota.

## 8 Analytical and methodological challenges in lipidomics

For many years, ensuring high data quality standards has become increasingly important for lipidomics, which has become one of the fastest-growing scientific disciplines. Good lipidomics practice should take the entire lipid workflow into consideration (Köfeler et al., 2021). However, there are several challenges in translating this type of lipidomic data into clinical practice. In the lipidomics workflow, each stage of the pre-analytical, analytical, and post-analytical processes can each have an impact on the results. In the pre-analytical stage, the study population, study design, individual differences, blood collection, and plasma/serum selection play important roles. In the analytical stage, lipid extraction methods, the mass spectrometry (MS) platforms used, and quality control materials may influence the results. In the post-analytical stage, data quality control, quantitation, data sharing, and the databases may affect the accuracy and comparability of the obtained data (Burla et al., 2018).

Biological samples can be obtained from plasma and serum, and less commonly from urine. However, various factors such as the immediate separation of plasma or serum, storage time and temperature before processing, transport duration, and the number of freeze–thaw cycles may affect the subsequent lipidomics analyses (Salihovic et al., 2023). In a study conducted on the plasma and serum lipidomics of healthy adults, plasma rather than serum was identified as the most suitable matrix for analyzing lipid biomarkers, as it better represents the original characteristics of an individual’s blood sample. In addition, repeated freeze–thaw cycles were reported to lead to decreases in the levels of most lipid metabolites and cause erroneous data (Ishikawa et al., 2014). In addition, the importance of blood sampling time in relation to blood collection is also emphasized (Burla et al., 2018). Inter and intra-day variations may lead to significant changes in certain lipid mediators. In one study, the most suitable sample preparation method for biological samples was determined to be liquid–liquid extraction performed using methanol/tert-butyl methyl ether (MeOH/MTBE). With this method, an extraction recovery of over 85% was achieved, while intra- and inter-day variation was shown to be below 15% (Rund et al., 2024).

Currently, various MS-based approaches are used for the analysis of plasma lipids, and there is no single method accepted as the standard in the field of lipidomics. Among the most commonly used methods are liquid chromatography coupled to mass spectrometry (LC–MS), direct flow injection, and direct-infusion/shotgun MS (DIMS). These methods may yield different results in terms of coverage, sensitivity, and specificity. However, within each approach, there are also significant differences in terms of methods and software depending on the characteristics of the MS instruments and/or the type of chromatography used (Burla et al., 2018). In addition, matrix effects are among the important analytical problems in lipidomic analyses. In LC–MS techniques, ion suppression is one of the matrix effects that can occur independently of the selectivity or sensitivity of the mass analyzer used. This situation may negatively affect the analytical performance parameters such as detection capacity, precision, and accuracy. Furthermore, the fact that the origin and underlying mechanisms of ion suppression have not yet been fully elucidated makes this issue more difficult to control (Volmer and Jessome, 2006).

In addition, matrix effects are among the important analytical problems in lipidomic analyses. Lipids may undergo hydrolysis, oxidation, or interspecies conversion through enzymatic or chemical processes during sample collection, preparation, processing, storage, and/or analytical procedures. Oxidation is also a major source of lipid degradation during these processes (Ulmer et al., 2021). In a study, the effects of different storage conditions and storage durations on plasma and serum lipidomics were analyzed. It has been reported that increased temperature and prolonged storage duration lead to degradation associated with hydrolysis and oxidation in certain lipid species. In particular, changes were observed in free fatty acids, diacylglycerols, and certain cholesteryl esters. In particular, changes were observed in free fatty acids, diacylglycerols, and certain cholesteryl esters (Reis et al., 2021).

Furthermore, it has been reported that the development of quantitative and highly reproducible protocols is important to ensure standardization in inter-laboratory lipidomics analyses (Ghorasaini et al., 2021). Indeed, in an inter-laboratory study conducted using frozen human plasma as a standard reference material, different results were observed among laboratories utilizing different lipidomics workflows (Bowden et al., 2017).

Due to all these challenges in lipidomics analyses, there is a need to develop standards in order to improve the reliability and comparability of lipidomics data (Lipidomics Standards Initiative Consortium, 2019; Initiative and L.S.). Accordingly, the Lipidomics Standards Initiative (LSI), which was established to address these challenges, aims to develop guidelines for core lipidomics workflows, including sample collection, storage, data processing, and reporting (Initiative and L.S.).

## 9 Conclusion and future perspectives

This review demonstrates that the microbiota–lipid–host axis is not merely a metabolic interaction network but also functions as a central regulatory system in the initiation, maintenance, and resolution of chronic inflammation. Microbiota-derived lipid metabolites, cell component-derived structural lipids, and functional lipid mediators shape the host metabolism and immune responses through multilayered, receptor-dependent, and context-specific signaling networks.

Across a broad pathological spectrum ranging from MetS and NAFLD to IBDs, neurodegenerative disorders, cancer, and chronic kidney disease, common mechanistic patterns emerge, including disrupted lipid mediator class switching, dominance of pro-inflammatory eicosanoids, insufficient resolution responses, and loss of intestinal barrier integrity. Alterations in the microbiota composition not only affect the metabolic substrate flux but also reprogram critical signaling pathways. Furthermore, the ability of the same lipid class to exert divergent or even opposing effects depending on the tissue type, concentration, and microenvironment underscores the highly dynamic and context-sensitive nature of this regulatory axis. Collectively, microbiota-shaped lipid mediator profiles play both initiating and modulatory roles in the pathogenesis of chronic inflammatory and metabolic diseases.

A deeper understanding of this axis may facilitate the development of novel therapeutic strategies that aim not only to suppress inflammation but also to restore physiological resolution mechanisms. Future research should focus on well-designed, large-scale, multi-center human studies to clarify the clinical relevance of microbiota–lipid–host interactions. The integration of lipidomics with other multi-omic approaches will be essential for elucidating the temporal and tissue-specific dynamics of microbiota-derived lipid mediators. Although personalized nutritional strategies and targeted probiotic/prebiotic interventions are promising, their efficacy and safety must be rigorously evaluated. Such an integrated perspective may contribute to the development of more precise and mechanism-based strategies for the management of chronic inflammation and metabolic disorders.

## Glossary

2-AG: 2-arachidonoylglycerol
AA: Arachidonic acid
ABCG5/8: ATP-binding cassette transport proteins G5 and G8
AEA: Anandamide
AgRP: Agouti-related peptide
ALA: α-linolenic acid
AMPK: AMP-activated protein kinase
Aβ: Beta amyloid protein
BA: Bile acid
BCFA: Branched-chain fatty acid
BSH: Bacterial bile salt hydrolase
CA: Cholic acid
CALA: Conjugated α-linolenic acid
CB: Cannabinoid receptor
CDCA: Chenodeoxycholic acid
CL: Cardiolipin
CLA: Conjugated linoleic acid
COX: Cyclooxygenase
CYP: Cytochrome P450
CYP7A1: Cholesterol 7α-hydroxylase
DC: Dendritic cell
DCA: Deoxycholic acid
DHA: Docosahexaenoic acid
DHET: Dihydroxyeicosatrienoic acid
DPA: Docosapentaenoic acid
ECS: Endocannabinoid system
EET: Epoxyeicosatrienoic acid
EPA: Eicosapentaenoic acid
FABP4: Fatty acid-binding protein 4
FASN: Fatty acid synthase
FFAR: Free fatty acid receptor
FGF19/15: Fibroblast growth factor 19/15
**FMO**_**3**_: Flavin monooxygenase 3
FXR: Farnesoid X receptor
GABA: Gamma-aminobutyric acid
GALT: Gut-associated lymphoid tissue
GLUT: Glucose transporter
GLP-1: Glucagon-like peptide-1
GPCR: G protein-coupled receptor
HDAC: Histone deacetylase
HETE: Hydroxy-eicosatetraenoic acid
HIF1: Hypoxia-inducible factor 1
HODE: Hydroxyoctadecadienoic acid
HPA: Hypothalamic–pituitary–adrenal
HYA: 10-hydroxy-cis-12-octadecenoic acid
HYB: 10-hydroxy-octadecanoic acid
HYC: 10-hydroxy-trans-11-octadekenoic acid
IAP: Intestinal alkaline phosphatase
IBD: Inflammatory bowel disease
IFN-γ: Interferon-gamma
IgA: Immunoglobulin A
IL: Interleukin
ILC: Innate lymphoid cell
IRAK: IL-1 receptor-associated kinase
JAK2: Janus kinase 2
KetoA: 10-oxo-cis-12-octadekenoic acid
KetoB: 10-oxo-octadecanoic acid
KetoC: 10-oxo-trans-11-octadekenoic acid
LA: Linoleic acid
LCA: Lithocholic acid
LDLR: Low-density lipoprotein receptor
LOX: Lipoxygenase
LPS: Lipopolysaccharide
LT: Leukotrien
LysoPS: Lyso phosphatidylserine
LysPC: Lyso phosphatidylethanolamine
MAPK: Mitogen-activated protein kinases
MCP-1: Monocyte chemoattractant protein-1
MDSC: Myeloid-derived suppressor cell
MetS: Metabolic syndrome
MS: Multiple sclerosis
mTORC1: Rapamycin complex 1
MUFA: Monounsaturated fatty acid
MyD88: Myeloid differentiation factor 88
NAFLD: Non-alcoholic fatty liver disease
NETs: Neutrophil extracellular traps
NF-κB: Nuclear factor kappa B
NKT: Natural killer T
NLRP3: Domain-like receptor family pyrin domain-containing 3
Nox2: Nicotinamide adenine dinucleotide phosphate oxidase 2
NPY: Neuropeptide Y
Nrf2: Nuclear factor erythroid 2-related factor 2
Olfr87: Olfactory receptor-87
oxoODE: Oxo-octadecadienoic acid
PD-1: Programmed cell death-1
PD-L1: Programmed cell death ligand-1
PGC-1α: Peroxisome proliferator-activated receptor gamma coactivator-1 alpha
PG: Prostaglandin
PIK3R3: Phosphoinositide-3-kinase regulatory subunit 3
**PLA**_**2**_: Phospholipase A_2_
PPAR-α: Peroxisome proliferator-activated receptor alpha
PPAR-γ: Peroxisome proliferator-activated receptor gamma
PUFA: Polyunsaturated fatty acid
ROS: Reactive oxygen species
RORγt: Retinoid-related orphan receptor
Rv: Resolvin
S1P: Sphingosine-1-phosphate
SCFA: Short-chain fatty acid
SFA: Saturated fatty acid
SHP: Small heterodimer partner
SIBO: Small intestinal bacterial overgrowth
SPM: Specialized pro-resolving mediator
SREBP2: Sterol regulatory element binding protein 2
STAT3: Signal transducer and activator of transcription 3
**T**_**3**_: Triiodothyronine
**T**_**4**_: Thyroxine
TAK1: Transforming growth factor-activated kinase 1
TF: Tissue factor
TGF-β: Transforming growth factor beta
TGR5: Takeda G protein-coupled receptor 5
Th17: T helper 17
TLR: Toll-like receptor
TMA: Oxidize trimethylamine
TMAO: Trimethylamine-N-oxide
TNF-α: Tumor necrosis factor-alpha
TRAF6: Tumor necrosis factor receptor-associated factor 6
Treg: Regulatory T-cells
TRX2: Thioredoxin-2
TUDCA: Tauroursodeoxycholic acid
TX: Thromboxane
UCP1: Uncoupling protein 1
UDCA: Urso-deoxycholic acid
VEGF: Vascular endothelial growth factor
VLDL: Very low-density lipoprotein
vWf: Von Willebrand factor
WPb: Weibel-Palade bodies
ZO-1: Zonula occludens-1
α-Syn: α-synuclein
